# Longitudinal analysis of the effects of cladribine tablets on B and T cell memory recall responses to vaccine antigens in people with multiple sclerosis

**DOI:** 10.3389/fimmu.2026.1881334

**Published:** 2026-10-22

**Authors:** Peter A. Csurhes, Pamela A. McCombe, Judith M. Greer

**Affiliations:** Frazer Institute, The University of Queensland, Brisbane, QLD, Australia

**Keywords:** antibodies, antigen-specific, cladribine tablets, multiple sclerosis, revaccination, T cells, vaccine memory responses

## Abstract

**Introduction:**

Cladribine tablets (CladT) are used as an immune reconstitution therapy for multiple sclerosis (MS). Patients are dosed in year 1 (time 0) and again after 12 months. The aim of therapy is to allow development of a reconstituted immune repertoire with reduced pathogenic autoreactivity. However, CladT could also affect the durability of memory T and antibody responses to bacterial and viral pathogens against which people with MS have previously been vaccinated. The objective of this study was to investigate changes in memory recall responses to vaccines over a 2-year period after the initial administration of CladT.

**Methods:**

Blood was collected prior to the initial administration of CladT and at 5 timepoints (3, 6, 12, 18 and 24 months) after administration from 18 people with MS. The effects of CladT on antigen-specific T cell and/or antibody responses to influenza, varicella zoster, tetanus toxoid, and SARS-CoV-2 were assessed.

**Results:**

While levels of antibodies remained relatively stable for most vaccine antigens, numbers of T cells specific for the vaccine antigens declined in most individuals, particularly after the second course of treatment. Observations from individuals revaccinated against influenza suggest that revaccination at 18–24 months after starting CladT treatment, after both courses have been given, can restore influenza-specific responses.

**Discussion:**

CladT treatment can cause a decline in numbers of vaccine-specific memory T cells. Numbers of influenza-specific memory T cells could be restored to pre-treatment levels by revaccination after the second course of treatment. Larger studies are needed to define antigen-specific revaccination schedules more broadly.

## 1 Introduction

Cladribine tablets (CladT) are an effective treatment for multiple sclerosis (1) and have the benefit of being a pulse therapy, where patients are given a short course of treatment in year 1 and again in year 2, after which there are long lasting effects on disease activity in most patients (2, 3). CladT is a chlorinated nucleoside analogue that can be phosphorylated by deoxycytidine kinase (present at high levels in lymphocytes) to produce tri-phosphorylated CladT, which interferes with DNA and RNA synthesis and is toxic to the cell (4). When used at standard doses in MS, treatment is followed by lymphopenia (1) with reduction of levels of T cells and B cells (5).

More detailed studies have shown that treatment is followed by prolonged depletion of memory B cells and decline in CD45RO^+^CD4^+^ memory T cells (6, 7), but there is overall little information on the functional effects of CladT on T cells or on antigen specific responses. CladT therapy is thought to reduce disease activity in MS by depleting lymphocytes involved in the pathogenesis of MS (8). However, it is likely that CladT therapy also depletes beneficial immune responses, including responses to vaccine antigens. There have been several studies investigating effects of CladT on levels of antibodies specific for SARS-CoV-2 vaccines (9–12), but few studies on other vaccine antigens (13–15) and none on T cell responses to these antigens. T cell responses are important, as they are essential for optimal cell-mediated immunity and long-lived antibody responses against pathogens, and, for some antigens, are more informative than antibody responses alone regarding the need for re-vaccination.

In the current study, we have investigated longitudinally, at 6 timepoints from pre-administration to 2 years after the initial administration of CladT, the effects on T cell and B cell subtypes, and on T cell and antibody responses to antigens from influenza, *Varicella zoster* (VZV), tetanus toxoid and SARS-CoV-2.

## 2 Methods

### 2.1 Subjects and sample collection

This study was approved by the Royal Brisbane & Women’s Hospital Human Research Ethics Committee (HREC/2020/QRBW/68192) and the University of Queensland HREC (2020002761). Twenty people with MS who were due to commence CladT therapy were recruited (between January 2020 and December 2022) and provided informed written consent. Antibody levels for VZV were tested prior to commencing CladT, and patients who did not have detectable levels of VZV-specific antibody were recommended to be vaccinated/revaccinated at least 6 weeks prior to commencement of CladT. Other than that, it was recommended that participants receive usual vaccinations (eg against influenza and SARS-CoV-2 (when they became available)), but this was not mandatory. Blood (40 mL) was collected prior to the first dose of CladT, and at 3, 6, 12, 18 and 24 months after the first dose. The “12 month” blood was collected before the second course of CladT treatment; if there was a delay in initiating the second course of treatment, all further blood samples were delayed. Eighteen individuals completed all 6 time points. Details of their demographics, prior treatments, washout periods, white cell counts prior to initiation of CladT, and relevant vaccination history (last 3 years prior to initiation of CladT and during study follow-up) are given in **Table 1**.

**Table 1:** Patient information.

Code	Sex	Age at MS onset	Age at CladT initiation	Prior medications (most recent)	Drug wash-out (months)	*WCC (x10^9^/L)	^§^Relevant vaccination/serology history (day, type & brand of vaccine)	Vaccinations (and infections) during the study	Vaccine-specific T cells present pre-CladT
CLAD01	F	35	49	Teriflunomide, **natalizumab**	1.5	4.2	Day -382, Flu (FluQuadri) Day -82, VZV (Varilrix) Day -82, DTP (Boostrix)	Day +186, SARS-CoV-2 (Comirnaty) Day +207, SARS-CoV-2 (Comirnaty) Day +267, SARS-CoV-2 (Comirnaty) Day +358, Flu (Vixigrip Tetra) Day +373, SARS-CoV-2 (Comirnaty) Day +764, Flu (FluadQuad)	Influenza (CD4^+^)
CLAD03	F	19	39	Copaxone, **alemtuzumab**	26	4.1	No vaccinations in 3 years prior to study **2016, Tested VZV IgG+**	No vaccinations during the study	Influenza (CD4^+^)
CLAD04	F	16	53	Fingolimod, **alemtuzumab**	51	5.1	Day -503, Flu (FluadQuad) Day -145, Flu (Fluarix Tetra) Day -29, SARS-CoV-2 (Comirnaty) **Day -16, Tested VZV IgG+** Day -7, SARS-CoV-2 (Comirnaty)	Day +156, SARS-CoV-2 (Comirnaty) Day +231, Flu (Vaxigrip Tetra)	Influenza (CD4^+^) VZV (CD8^+^)
CLAD05	M	18	36	**Alemtuzumab**	29	7.4	Day -128, SARS-CoV-2 (Comirnaty) Day -104, SARS-CoV-2 (Comirnaty) **Day -17, Tested VZV IgG+**	Day +145, SARS-CoV-2 (Comirnaty)	Influenza (CD4^+^) VZV (CD4^+^, CD8^+^) Tet tox (CD4^+^)
CLAD06	F	40	60	nil	n/a	6.9	Day -1056, DTP (Boostrix) Day -146, SARS-CoV-2 (Vaxzevria) Day -79, Flu (AfluriaQuad) Day -63, SARS-CoV-2 (Vaxzevria) Day -38, VZV (Zostavax) **Day -7, Tested VZV IgG+**	Day +80, SARS-CoV-2 (Comirnaty) Day +267, Flu (FluQuadri) Day +316, SARS-CoV-2 (Comirnaty) Day +605, SARS-CoV-2 (Comirnaty Bivalent BA. 4-5) Day +605, Flu (Vaxigrip Tetra)	Influenza (CD4^+^) VZV (CD4^+^) Tet tox (CD4^+^)
CLAD07	F	34	47	IFN-β1a, pegylated IFN-β1a, **dimethyl fumarate**	0.25	7.7	Day -115, SARS-CoV-2 (Vaxzevria) Day -31, SARS-CoV-2 (Vaxzevria)	Day +76, SARS-CoV-2 (Comirnaty) Day +175, SARS-CoV-2 (Spikevax) Day +294, SARS-CoV-2 (Spikevax) Day +300, Flu (Vaxigrip Tetra) Day +650, Flu (FluadQuad)	Influenza (CD4^+^)
CLAD09	F	21	36	Natalizumab, **Copaxone**	0.5	5.7	Day -835, DTP (Adacel) Day -536, Flu (generic influenza) Day -184, Flu (Vaxigrip Tetra) Day -184, DTP (Boostrix) Day -80, SARS-CoV-2 (Comirnaty) Day -51, SARS-CoV-2 (Comirnaty) **Day -11, Tested VZV IgG+**	Day +74, SARS-CoV-2 (Spikevax) Day +180, SARS-CoV-2 (Spikevax) Day +213, Flu (Vaxigrip Tetra) Day +530, Flu (FluadQuad)	Influenza (CD4^+^) VZV (CD4^+^) Tet tox (CD4^+^)
CLAD10	F	33	49	**Copaxone**	2	**8.9**	**Day -234, tested VZV IgG negative** Day -144, SARS-CoV-2 (Vaxzevria) Day -60, SARS-CoV-2 (Vaxzevria) Day -27, DTP (Adacel) Day -25, VZV (Zostavax) Day -25, Flu (Vaxigrip Tetra)	Day +93, SARS-CoV-2 (Comirnaty) Day +192, Flu (Afluria Quad) **Day +492, Covid infection**	Influenza (CD4^+^) VZV (CD4^+^)
CLAD12	F	30	30	nil	n/a	6.1	No vaccinations in 3 years prior to study **Day -6, VZV IgG+**	Day +105, Flu (Vaxigrip Tetra) Day +481, Flu (FluadQuad)	Influenza (CD4^+^)
CLAD15	M	44	49	**Natalizumab**	1	7.9	Day -1093, Flu (FluarixTetra) Day -755, Flu (AfluriaQuad) Day -342, SARS-CoV-2 (Vaxzevria) Day -317, Flu (AfluriaQuad) Day -258, SARS-CoV-2 (Vaxzevria) Day -97, SARS-CoV-2 (Comirnaty) **Day -93, Tested VZV IgG+** Day -24, Flu (FluQuadri)	Day +82, SARS-CoV-2 (Comirnaty) **Day +91, Covid infection** Day +358, Flu (Afluria Quad) Day +358, SARS-CoV-2 (Spikevax Bivalent BA. 4-5)	Influenza (CD4^+^) VZV (CD4^+^)
CLAD16	F	23	41	IFN-β1a, **dimethyl fumarate, methotrexate**	1	4.6	Day -1056, Flu (FluarixTetra) Day -278, Flu (Vaxigrip Tetra) Day -262, SARS-CoV-2 (Comirnaty) Day -262, SARS-CoV-2 (Comirnaty) Day -130, SARS-CoV-2 (Comirnaty) **Day -8, Tested VZV IgG+**	Day +14, Flu (AfluriaQuad)	Influenza (CD4^+^)
CLAD17	F	23	33	nil	n/a	6.2	Day -1050, Flu (AfluriaQuad) Day -1050, DTP (Boostrix) Day -780, DTP (Adacel) Day -720, Flu (generic influenza) Day -189, SARS-CoV-2 (Comirnaty) Day -168, SARS-CoV-2 (Comirnaty) Day -29, Varicella (Varivax)	Day +363, Flu (AfluriaQuad)	Influenza (CD4^+^) VZV (CD4^+^)
CLAD19	F	28	61	Teriflunomide, pegylated IFN-β1a, **Copaxone**	0.5	13.8	Day -1077, Flu (FluQuadri) Day -707, Flu (Vaxigrip Tetra) Day -398, Flu (FluarixTetra) Day -376, SARS-CoV-2 (Vaxzevria) Day -292, SARS-CoV-2 (Vaxzevria) Day -137, SARS-CoV-2 (Comirnaty) Day -46, Flu (Influvac tetra) **Day -32**, **Tested VZV IgG+** Day -25, DTP (Adacel)	Day +80, SARS-CoV-2 (Comirnaty) Day +346, SARS-CoV-2 (Spikevax Bivalent BA. 1) Day +358, Flu (Vaxigrip Tetra) Day +690, Flu (AfluriaQuad)	Influenza (CD4^+^) VZV (CD4^+^)
CLAD20	M	56	59	**Teriflunomide**	1.25	4.7	Day -297, SARS-CoV-2 (Vaxzevria) Day -211, SARS-CoV-2 (Vaxzevria) **Day -28**, **Tested VZV IgG+** No other vaccinations since childhood	**Day +10**, **Covid infection**	Tet tox (CD4^+^)
CLAD21	F	50	61	**Teriflunomide**	0.25	4.1	Day -772, Flu (Vaxigrip Tetra) Day -397, Flu (Flu Quadri) Day -284, SARS-CoV-2 (Vaxzevria) Day -242, SARS-CoV-2 (Vaxzevria) Day -118, SARS-CoV-2 (Comirnaty)	No vaccinations during the study	Influenza (CD4^+^) VZV (CD4^+^)
CLAD24	M	47	47	nil	n/a	5.5	Day -60, Flu (generic influenza)	Day +301, Flu (Vaxigrip Tetra) Day +570, Flu (Vaxigrip Tetra)	Influenza (CD4^+^) Tet tox (CD4^+^)
CLAD26	F	21	44	**Natalizumab**	1	9.0	**Day -33, Tested VZV IgG+**	No vaccinations during the study	Influenza (CD4^+^) VZV (CD4^+^, CD8^+^)
CLAD27	F	23	46	Fingolimod, **natalizumab**	0.75	4.4	Day -529, SARS-CoV-2 (Comirnaty) Day -503, SARS-CoV-2 (Comirnaty) **Day -84, Tested VZV IgG+**	Day +300, Flu (Vaxigrip Tetra)	Influenza (CD4^+^) VZV (CD8^+^)

Bold underlined elements in the Table refer to the bold underlined point in the heading of the same column (e.g. in the Prior medications column, the most recent medications are in bold underlined font)*WCC – White cell count – tested immediately prior to CladT initiation. Normal range is 4.0-11.0 x10^9^ cells/L.

§Vaccine information:

Influenza vaccines: All quadrivalent vaccines (2 x A + 2 x B strains), with most being egg-based vaccines (Vaxigrip Tetra, Fluarix Tetra, Afluria Quad, and FluQuadri) and one adjuvanted vaccine (Fluad Quad).

SARS-CoV-2 vaccines: Comirnaty – SARS-CoV-2 spike protein (mRNA) (Pfizer); Vaxzevria – ChAdOx1-S [recombinant] (AstraZeneca); Spikevax – produced by Moderna.

DTP (diptheria/tetanus/pertussis) vaccines: Adacel and Boostrix.

VZV vaccines: All live-attenuated vaccines (Varivax, Varilrix, Zostavax).

From the blood samples, 5 mL blood was allowed to clot for serum, and the remainder was used for cell-based assays, as outlined below. In addition to the blood samples taken for this study, blood was also collected for routine haematology measurements, including T cell and B cell numbers, as part of the clinical care of the study participants.

### 2.2 Measurement of lymphocyte numbers

A 50μL aliquot of whole fresh blood at each blood collection timepoint was used immediately for whole blood count flow cytometry using BD Trucount tubes to calculate cell numbers in each major cellular subset per mL of patient blood. Absolute numbers of major cell subtypes (lymphocytes, B cells, T cells, neutrophils, monocytes, and natural killer cells) were counted for each participant at each timepoint. Antibodies used in the flow cytometry panel are shown in **Supplementary Table S1** and the flow cytometry gating strategy is shown in **Supplementary Figure S1a**. Calculations of cells per mL of blood were done according to the BD Trucount tube instructions. This information was required for accurate enumeration of antigen-specific cells per mL of patient blood.

### 2.3 Enumerating T cell responses to antigens

Peripheral blood mononuclear cells (PBMC) collected at each timepoint were prepared from each blood sample and cryopreserved for subsequent batch-testing of vaccine-antigen-specific T cell responses. Batch testing cells that have been collected at multiple timepoints from the same individual reduces some of the variation that could potentially be introduced if each sample was tested separately.

Antigens to be used were initially tested with PBMC from 4 healthy controls to determine optimal concentrations for use. The antigens and final concentrations used in the assays were: tetanus toxoid (5µg/mL, List Biologicals), the 2020 quadrivalent influenza vaccine (1µg/mL, Fluquadri SANAV7740), *Varicella zoster* virus (VZV) glycoprotein E peptide pool (1 µg/mL, JPT Peptide Technologies), keyhole limpet haemocyanin (KLH (10 µg/mL, Merck KGaA, Darmstadt, Germany)) as a non-relevant antigen, and phorbol myristate acetate/Ionomycin C (BD) as a positive control for T cell studies. PBMC were thawed, rested for one day, and then stimulated with the antigens overnight, with Golgi-blocking agents brefeldin A and monensin (BD) added to cause cytokines induced by the antigen stimulation to accumulate in the cells.

Following activation, PBMC were surface stained with a fixable viability dye (Live/dead Aqua, Invitrogen) and fluorophore-labelled antibodies (listed in **Supplementary Table S1**) to allow separation of cells into CD4^+^ or CD8^+^ naïve, central memory (T_CM_), effector memory (T_EM_), or effector memory re-expressing CD45RA (T_EMRA_) T cells. The cells were then fixed, permeabilized and labelled with antibodies against interferon gamma (IFNγ), tumour necrosis factor (TNF) and interleukin 2 (IL-2) and assessed by flow cytometry (**Supplementary Figure 1b**) on a 10 color Gallios cytometer (Beckman Coulter, CA, USA). More than 200, 000 events were collected to enable accurate estimation of antigen-specific T-cell frequencies. Flow cytometry data were analysed using Kaluza 1.3 software (Beckman Coulter).

For each measurement of the frequency of antigen-specific cytokine-producing T cells, the background frequency of cytokine production in the absence of antigen was subtracted to yield a background corrected frequency. The information from these assays, combined with the whole blood counts, was used to calculate the numbers of vaccine-antigen specific T cells per mL of patient blood. A positive antigen specific response was one where the number of cells in the antigen stimulated population was both at least twice the number of cells in the no antigen stimulated wells and greater than the mean ± 2 standard deviations of the unstimulated wells for all timepoints tested for each individual. A threshold of 40 cells was set as the minimum number of antigen-specific cells that had to be present in order to record a “detectable” number of cells; this was >2 times the maximum number of KLH-positive cells found in any well. T cells producing IL-2, IFN-γ or TNF were generally found in the T_EM_ or T_EMRA_ subsets of the T cells.

### 2.4 Antibody responses

Serum from each donor at each timepoint was collected and stored at -80 °C for use in measurement of vaccine-induced antigen-specific antibody responses. For consistency of results, all serum samples (at the 6 time points) from an individual were tested in duplicate at the same time for IgG antibody responses.

Commercially available ELISA kits for detection of IgG specific for influenza A (My BioSource, San Diego CA), Human VZV (MyBioSource), VaccZyme™ tetanus toxoid (The Binding Site, Birmingham, UK), and the SARS-CoV-2 receptor binding domain (RBD, a target of protective antibodies induced by most of the Covid-19 vaccines) (Invitrogen), were used to measure levels of IgG for the different antigens. When the study commenced, the Covid-19 pandemic had not begun, and insufficient blood was being collected to do T cell testing against SARS-CoV-2 antigens; however, since serum samples were available at all timepoints and all except one of the patients in the study were vaccinated against SARS-CoV-2 during the course of the study, antibodies against the SARS-CoV-2 RBD were also measured.

### 2.5 Statistical analyses

For comparisons of changes in numbers of cells or levels of antibodies at the five timepoints following CladT administration across the whole group of patients, the pre-CladT sample number for each measurement was given a value of 100%, and the remaining samples are reported as a percentage of that. The pre-CladT numbers for each measure in each patient can be found in **Supplementary Figure S2**. The percentages of cells of different subtypes were compared to the pre-CladT baseline measurements using the Friedman test (repeated measures test for non-parametric data), with Dunn’s correction for multiple comparisons. For comparison of the effects of revaccination at different timepoints on influenza-specific responses, a mixed effects model using multiple comparisons and comparison of groups within each timepoint was used. All analyses were performed using GraphPad Prism 10.4.1. *P* < 0.05 was considered to be significant. In reporting *P* values throughout the paper, * indicates *P* < 0.05; ** indicates *P* < 0.01; *** indicates *P* < 0.001; **** indicates *P* < 0.0001.

## 3 Results:

### 3.1 Overview of changes in blood cell types and numbers

Apart from neutrophils, all other blood cell types measured (lymphocytes, T cells, B cells, monocytes and NK cells) were significantly depleted (compared to pre-CladT timepoint) at one or more timepoints after treatment with CladT (**Figure 1**). By the end of the 24-month follow-up, median numbers of all cell types, apart from CD3^+^ T cells and monocytes, had returned to close to baseline numbers. 

**Figure 1:**
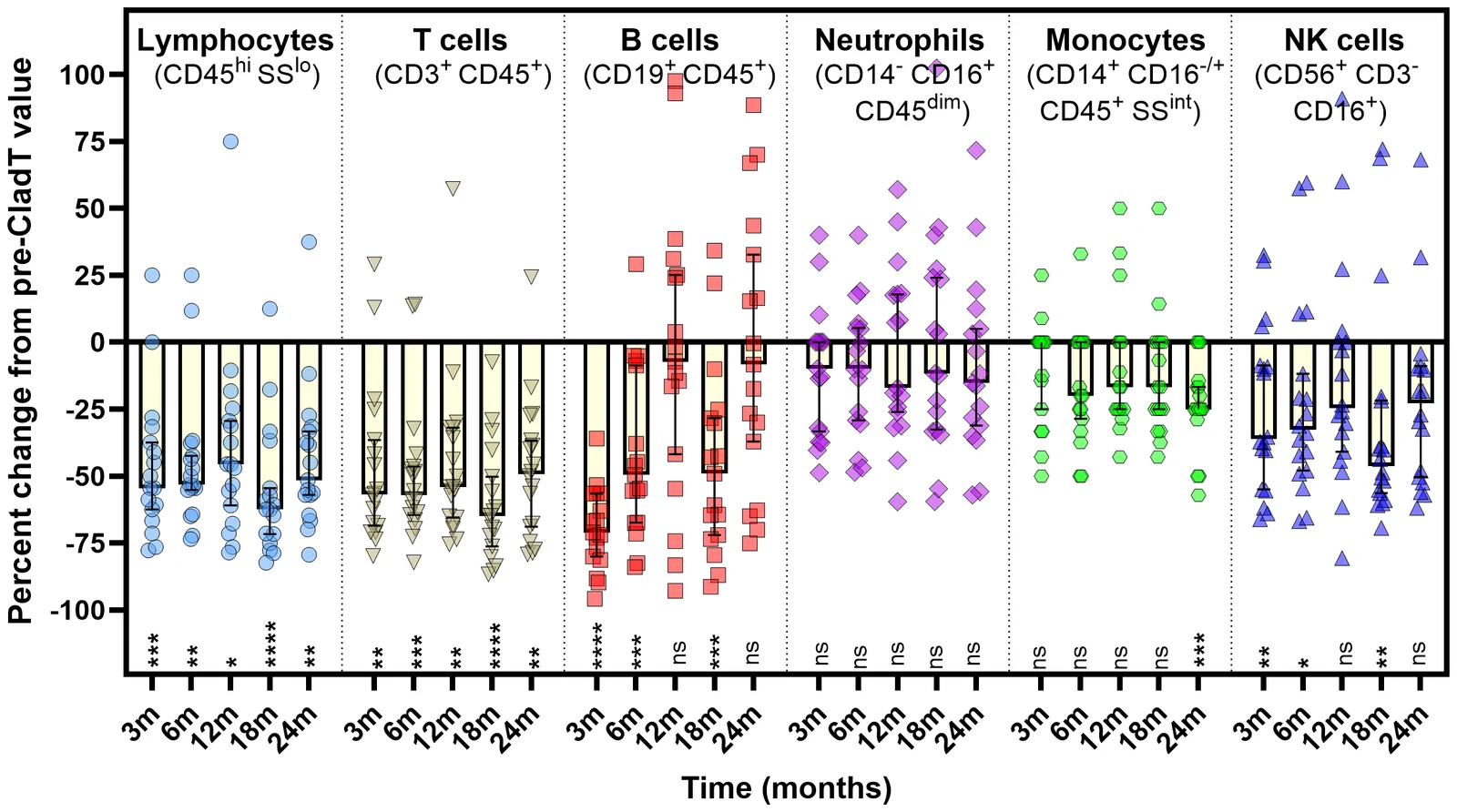
Percent change in numbers of lymphocytes, T cells, B cells, neutrophils, monocytes and NK cells (per mL of blood), compared to pre-CladT numbers. Symbols represent individual patients and bars and error bars represent the median and interquartile range of the percent change values. The pre-CladT timepoint is represented by the line at 0 in this figure, so symbols below the line at 0 represent a decrease in the number of cells from baseline, and those above the 0 line represent an increase in the numbers of cells/mL blood compared to baseline. Statistical analysis: Friedman test with Dunn’s correction for multiple comparisons. Timepoints significantly different from the pre-CladT value are indicated by the number of asterisks (****P<0.0001; ***P<0.001; **P<0.01; * P<0.05; ns, not significant).

More in-depth analysis of CD3^+^ T-cell subsets (**Figure 2**) showed that the overall patterns of change for CD4^+^ and CD8^+^ naïve and effector memory re-expressing CD45RA (T_EMRA)_ T cell subsets reflect the findings for total T-cell numbers, with generally consistent changes among individuals. However, the central memory (T_CM_) and effector memory (T_EM_) cells, particularly in the CD8^+^ subset, show less depletion than the other cell subsets, and considerable variability amongst individuals, with some patients showing a large increase in the cell numbers per mL of blood, even at the 3-month timepoint (**Figure 2**).

**Figure 2:**
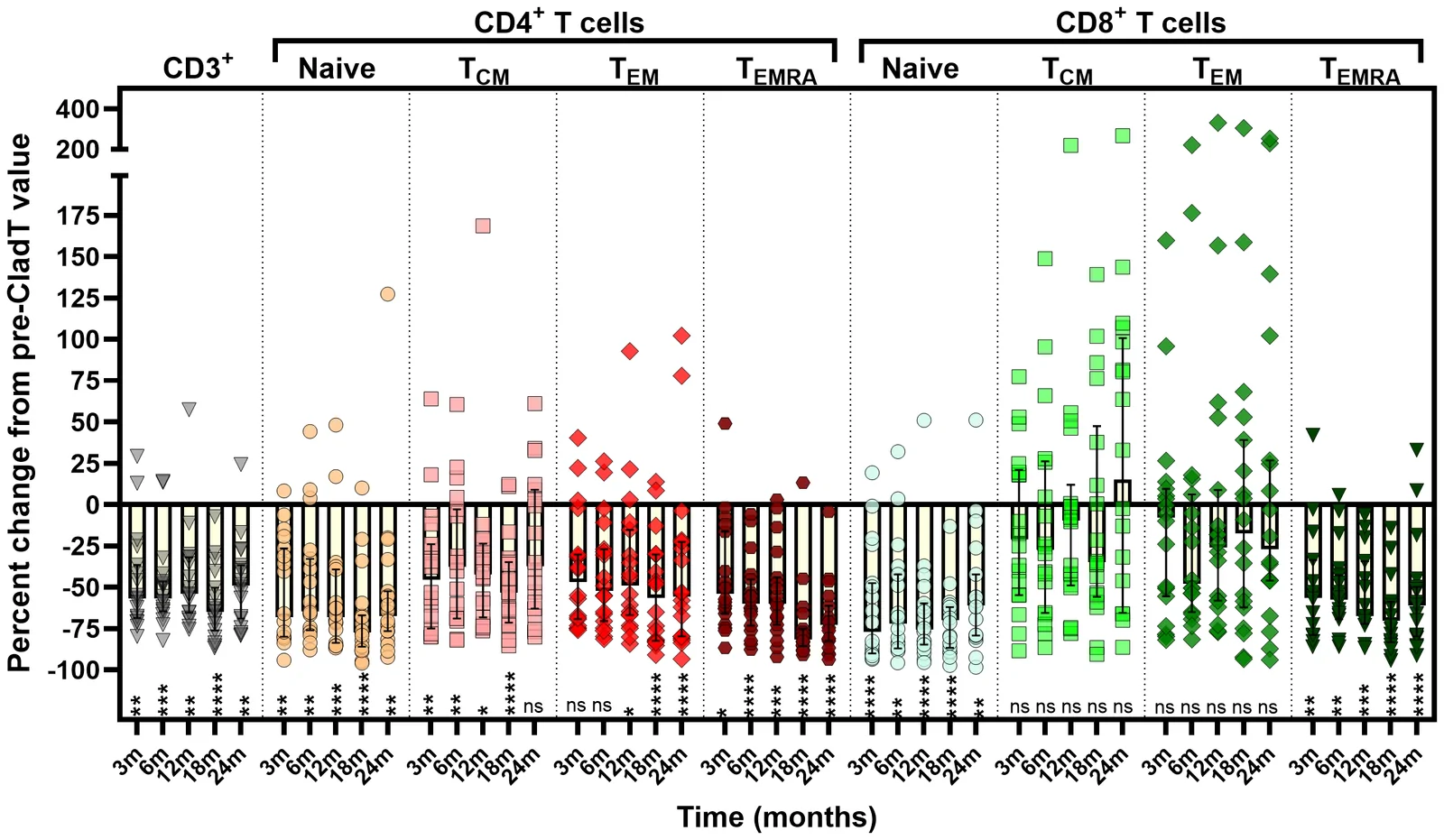
Percentage change in T cell subset numbers (per mL of blood) from pre-CladT values. CD4^+^ and CD8^+^ T cells were further subdivided into 4 memory subsets, based on staining for expression of CD45RA and CD62L [CD45RA^+^CD62L^+^ naïve, CD45RA^-^CD62L^+^ central memory (T_CM_), CD45RA^-^CD62L^-^ effector memory (T_EM_) and CD45RA^+^CD62L^-^effector memory re-expressing CD45RA (T_EMRA_)]. Each individual patients’ results are indicated by a symbol. Bars and error bars represent the median and interquartile range of the percent change values. The pre-CladT timepoint is represented by the line at 0 in this figure, so symbols below the 0 line represent a decrease in the number of cells from baseline, and those above the 0 line represent an increase in the numbers of cells/mL blood compared to baseline. Statistical analysis: Friedman test with Dunn’s correction for multiple comparisons. Timepoints significantly different from the pre-CladT value are indicated by the number of asterisks (****P<0.0001; ***P<0.001; **P<0.01; * P<0.05; ns, not significant).

By the end of the 2-year period, 50% of patients had B cell levels equal to or higher than the pre-CladT level (**Figure 3A**). However, there were marked differences between percent changes in naïve and memory B cells following CladT treatment. Naïve B cells rapidly returned to higher than pre-CladT levels in 50% of the patients, with a clear correlation (r^2^ = 0.89; *P* < 0.0001) between the numbers of naïve B cells present at the 12-month time-point and the final levels at 24 months (**Figure 3B**). In contrast, memory B cells showed a sustained decrease at all timepoints, with only 3 individuals recovering memory B cell levels to > 50% of pre-CladT levels by the 24-month timepoint; most patients had levels of around 10-20% of pre-CladT levels (1 patient had only 1.3%) at the 24-month timepoint (**Figure 3A**). CD19^+^ CD138^+^ cells (most likely short-lived plasmablasts, although this population could also contain some longer-lived plasma cells) initially dropped significantly in most patients. Recovery of these cells was variable from one person to another.

**Figure 3:**
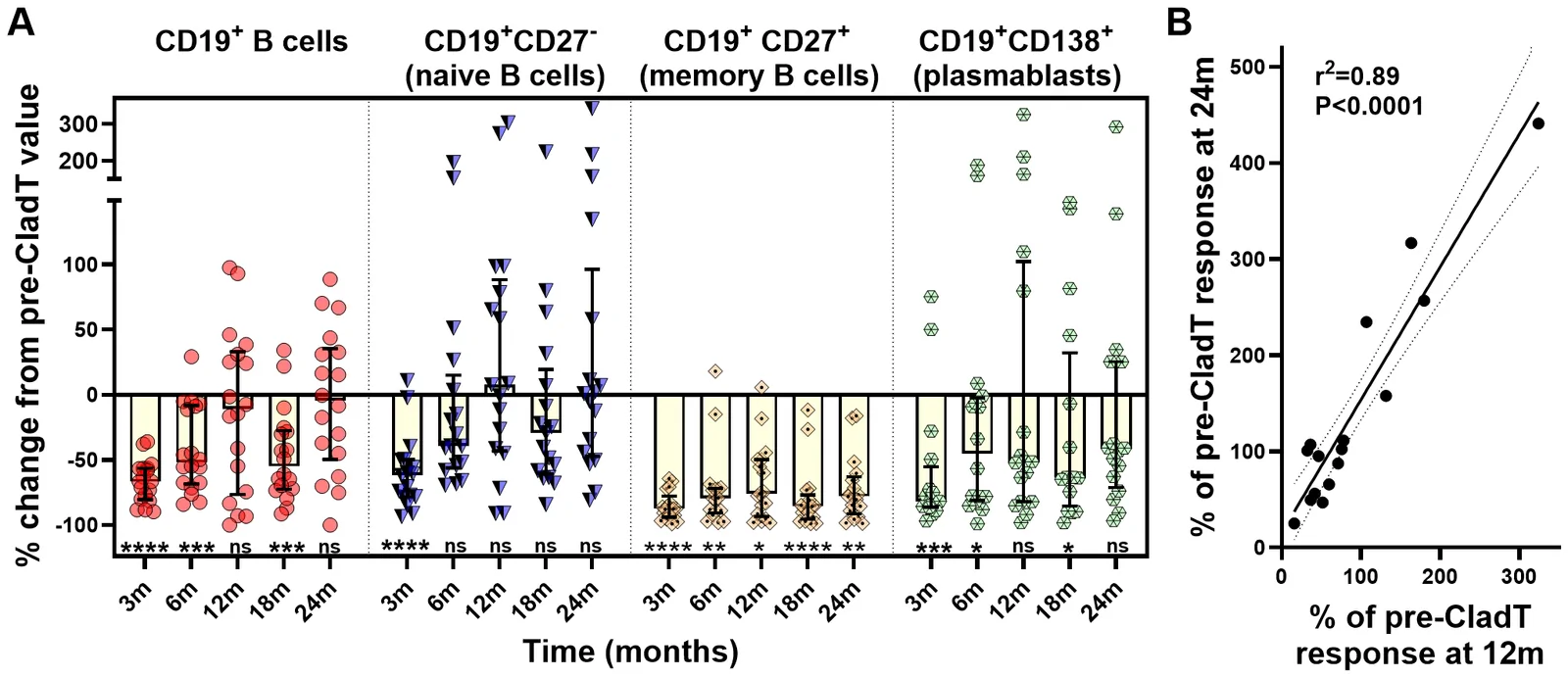
**(A)** Percentage change in B cell subset numbers (per mL of blood) from pre-CladT values. Each individual patients’ results are indicated by a symbol. Bars and error bars represent the median and interquartile range of the percent change values. The pre-CladT timepoint is represented by the line at 0 in this figure, so dots below the 0 line represent a decrease in the number of cells from baseline, and those above the 0 line represent an increase in the numbers of cells/mL blood compared to baseline. Statistical analysis: Friedman test with Dunn’s correction for multiple comparisons. Timepoints significantly different from the pre-CladT value are indicated by the number of asterisks (****P<0.0001; ***P<0.001; **P<0.01; * P<0.05; ns, not significant). **(B)** There was a strong positive correlation between the percent of pre-CladT response for naive B cells at twelve and twenty-four months.

### 3.2 Antigen-specific immune responses to vaccine antigens following CladT treatment

Full details of T cell and antibody responses to each of the vaccine antigens in each of the patients, along with baseline (pre-CladT) numbers of cells or antibody concentration, are shown in Panels A and B, respectively, of figures in **Supplementary Figure S2**. At the pre-CladT timepoint, there was evidence of influenza-specific T cells in 17/18 patients (median of 245 cells/mL blood), VZV-specific T cells in 11/18 patients (median of 137 cells/mL blood), and tetanus toxoid specific cells in 5 of the 18 patients (median of 130 cells/mL blood). Antibodies specific for influenza, VZV, tetanus and SARS-CoV-2 RBD were detectable in all individuals. The effects of CladT treatment on these cells and antibodies is described below.

#### 3.2.1 Influenza-specific T cell and antibody dynamics and revaccination-associated restoration

Most individuals (17/18) showed a CD4^+^ T cell response to influenza at the pre-CladT timepoint, which overall dropped off significantly after the first dose of CladT (**Figure 4A**). There were almost no CD8^+^ influenza-specific cytokine-producing cells detected in our assays, likely due to the nature of the influenza antigen used to stimulate cells, which would have been more likely to activate CD4^+^ than CD8^+^ T cells. **Supplementary Figures S3A and S3B**, show the changes in the absolute number of influenza specific CD4^+^ T cells/mL blood in each of the 17 individuals who made an influenza-specific T cell response at each timepoint. Thirteen of the 17 individuals who carried T cells specific for influenza subsequently received one or more vaccinations with the Australian quadrivalent influenza vaccine current for the year(s) during which this study was being done (between 2021 – 2024), all of which contained 2 type A and two type B strains. Of those who received vaccinations, 7 were vaccinated between 6–12 months and then again between 18–24 months after CladT was initiated and the other 6 individuals were only vaccinated between 6–12 months after CladT was initiated. Vaccination between 6–12 months typically induced an increase in influenza-specific T cells in most individuals, but this dropped off again after the second course of cladribine. Revaccination between 18–24 months restored the numbers of influenza-specific T cells to pre-cladribine levels (**Figure 4B**). Vaccinations occurring only during 6–12 months after the initial CladT treatment and not after the second course of CladT resulted in numbers of influenza-specific T cells that were not significantly higher than those in individuals who were not vaccinated during the study (**Figure 4B**; **Supplementary Figures S3A, B**). Individuals who were not vaccinated against influenza during the study (CLAD03, CLAD05, CLAD21 and CLAD26) had the lowest numbers of influenza specific T cells at the end of the study (**Figure 4B**); indeed, in the pre-CladT sample, CLAD26 had the highest frequency of influenza-specific T cells in their blood of any of the subjects (1, 488 cells/mL), but without any vaccinations throughout the course of the study, by 24 months, they had the lowest frequency (<40 cells/mL, i.e. below the limit of detection).

**Figure 4:**
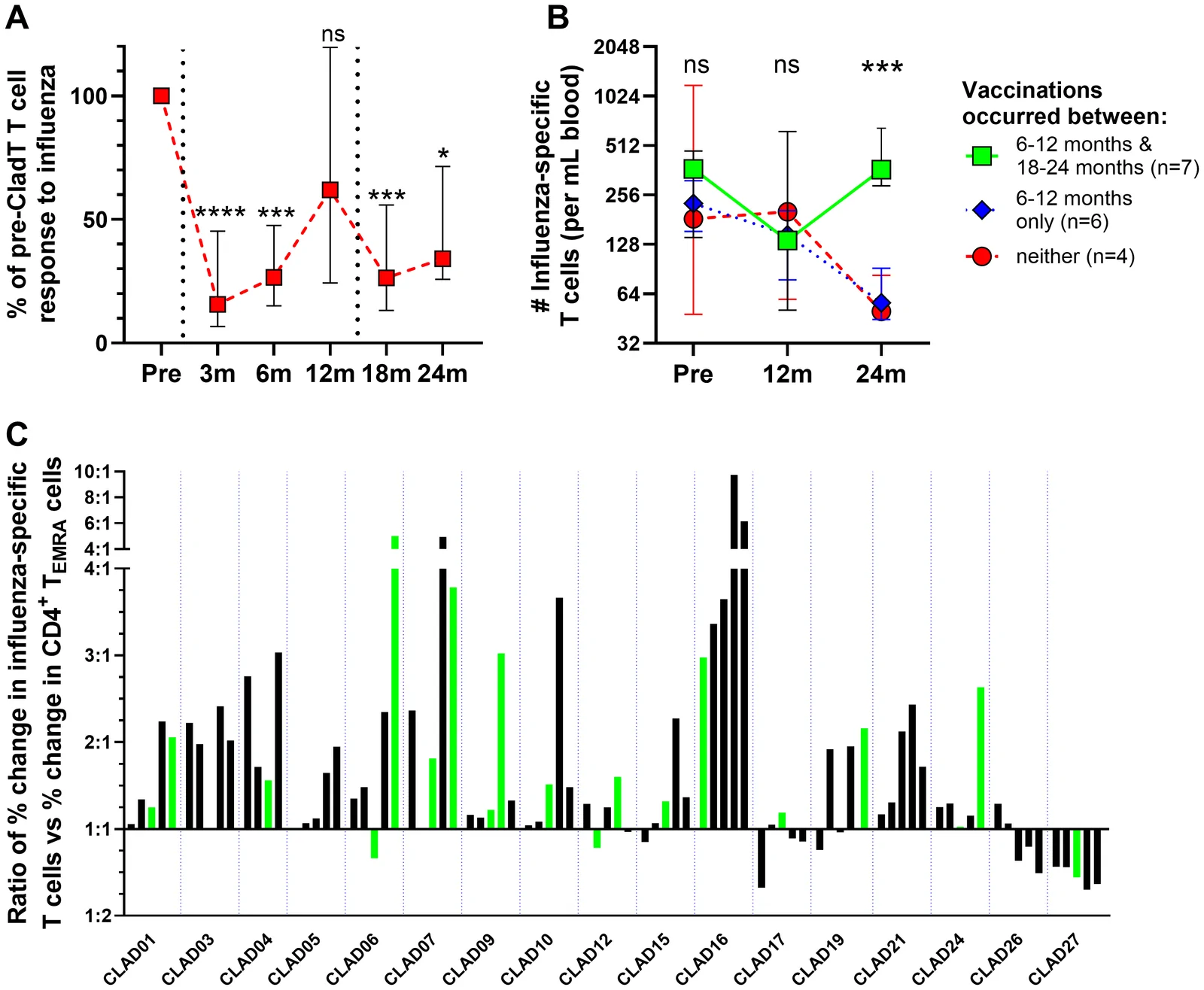
Changes in numbers of CD4^+^ T cells specific for influenza following CladT therapy. **(A)** Overview of changes in percentages of influenza-specific CD4^+^ T cells, compared to the pre-CladT level (Pre), at the 3-month (3m), 6-month (6m), 12-month (12m), 18-month (18m) and 24-month (24m) timepoints. Each symbol and error bar represents the median and interquartile range of the 17 patients who had pre-existing influenza-specific cells. The vertical dotted lines represent the timing of the two treatments with CladT relative to the blood collection timepoints. Statistical analysis: Friedman test with Dunn’s correction for multiple comparisons **(B)** Influence of timing of revaccinations on numbers of influenza-specific T cells/mL of blood at the Pre, 12m and 24m timepoints. Patients vaccinated at both the 6–12 and 18–24 month timepoints had significantly more influenza-specific CD4^+^ T cells in their blood at the 24 m timepoint than patients only vaccinated between months 6–12 and unvaccinated patients. Statistical analysis: mixed effects model using multiple comparisons and comparison of groups within each timepoint. **(C)** Changes in numbers of influenza-specific CD4^+^ T cells are independent of changes in numbers of CD4^+^ T_EMRA_ cells in most patients. For each patient, 5 bars are shown that represent the ratio of the % change from baseline of the influenza-specific cells to the % change from baseline of the total CD4^+^ T_EMRA_ cells at each timepoint (3m, 6m, 12m, 18m and 24m). If the influenza-specific cells only changed due to changes in total numbers of CD4^+^ T_EMRA_ cells, then the bar would be at the line at 1:1. Green bars represent timepoints where there had been a vaccination just prior to that timepoint. Statistical analysis: Friedman test with correction for multiple comparisons by controlling the false discovery rate (overall P<0.0001, indicating that the typical patient does not stay at a 1:1 ratio). For **(A, B)**, compared to pre-CladT timepoint, ****P<0.0001, ***P<0.001, *P<0.05, ns, not significant.

To evaluate whether the changes in numbers of influenza reactive cells were correlated with the changes in the total numbers of T cells in each subset, we also compared the ratio of changes in influenza-specific T cells vs the changes in CD4^+^ T_EM_ and CD4^+^ T_EMRA_ cells (the subsets with the highest frequency of antigen-specific CD4^+^ T cells) at each timepoint (**Figure 4C** and **Supplementary Figure S3C**). The ratio differed between individuals, but in most had moved away from the 1:1 ratio that would be expected if the observed changes in numbers of influenza-specific cells were only due to the depletion of specific cell type. Analysis of the pairs of data using the Friedman test for repeated measures with correction for multiple comparisons by controlling the False Discovery Rate (FDR) gave a P<0.0001 for CD4^+^T_EMRA_ (**Figure 4C**) and P = 0.0037 for CD4^+^T_EM_ (**Supplementary Figure S3C**) and confirmed that the typical patient does not stay at a 1:1 ratio. This strongly suggests that changes seen in influenza-specific CD4^+^ T_EMRA_ or CD4^+^T_EM_ cells from the majority of individuals are not merely due to the effects of CladT on T cell numbers overall.

Overall levels of antibody to influenza did not change significantly throughout the course of the study, compared to baseline levels (**Figure 5A**), although the large error bars in antibody responses to influenza reflect the variations in the number and timing of vaccinations amongst the group. However, the dynamic range of antibodies did vary quite markedly from one person to the next (**Figure 5B**). The assay used to detect influenza-specific antibody levels was calibrated to determine if the antibody levels are likely to be protective. Five patients had levels below the protective level against influenza at one or more timepoints during the study (**Figure 5B**).

**Figure 5:**
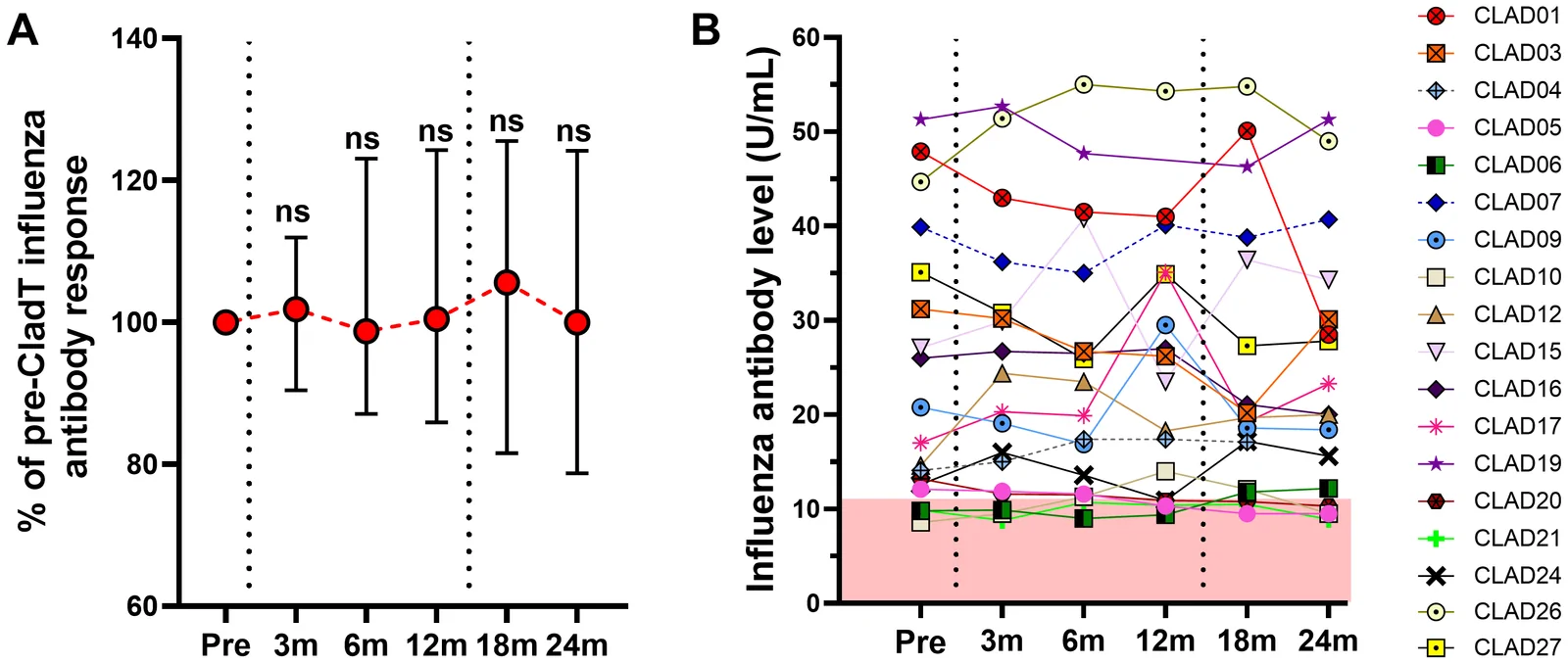
Influenza-specific antibody responses in all 18 subjects in the study. **(A)** There were no significant changes at the group level in overall amounts of influenza-specific antibody, compared to the pre-cladribine levels. The symbol indicates the median response, with the error bars showing the interquartile range. **(B)** The influenza-specific antibody levels (in U/mL) for each patient. The vertical dotted lines indicate when each course of CladT was given. The red shading at the bottom of the graph indicates the cut-off for protective levels of antibodies (11 U/mL). Statistical analysis for A: Friedman test with Dunn’s correction for multiple comparisons; ns, not significant.

#### 3.2.2 VZV-specific T cell and antibody responses in some patients increased following CladT treatment

For VZV responses, 14/18 patients had existing anti-VZV antibodies and were not revaccinated prior to commencing CladT. The other four were vaccinated against VZV just prior to starting CladT (**Table 1**). Nine of the patients had detectable levels of VZV-specific CD4^+^ T cells and 4 had detectable levels of VZV-specific CD8^+^ T cells at the pre-CladT (**Table 1**) timepoint. None of the patients showed overt signs of shingles during the 2-year follow-up period. Five of the 9 individuals with detectable CD4^+^ responses showed increased levels of VZV-specific T cells either at the 3- or 18-month timepoints, i.e. at the first blood collection timepoints after the first or second rounds of treatment with CladT, despite none of them being revaccinated during the study (**Figure 6A**; **Supplementary Figure S2**). This transient increase in cell numbers was not seen in the 4 individuals with detectable numbers of VZV-specific CD8^+^ T cells at the pre-CladT timepoint. Instead, in those 4, VZV-specific CD8^+^ T cell numbers rapidly dropped to undetectable levels and remained that way until the end of the study (**Figure 6B**). As with influenza specific CD4^+^ T cells, analysis of the ratio of percent changes in VZV-specific CD4^+^ T cells vs percent changes in CD4^+^ T_EM_ and CD4^+^ T_EMRA_ cells showed significant divergence from a 1:1 ratio of change, again suggesting that the differences seen in VZV-specific CD4^+^ T numbers are not just due to the effects of CladT on T cell numbers overall. Numbers of patients with VZV-specific CD8^+^ T cells (n=4) were insufficient for this type of analysis.

**Figure 6:**
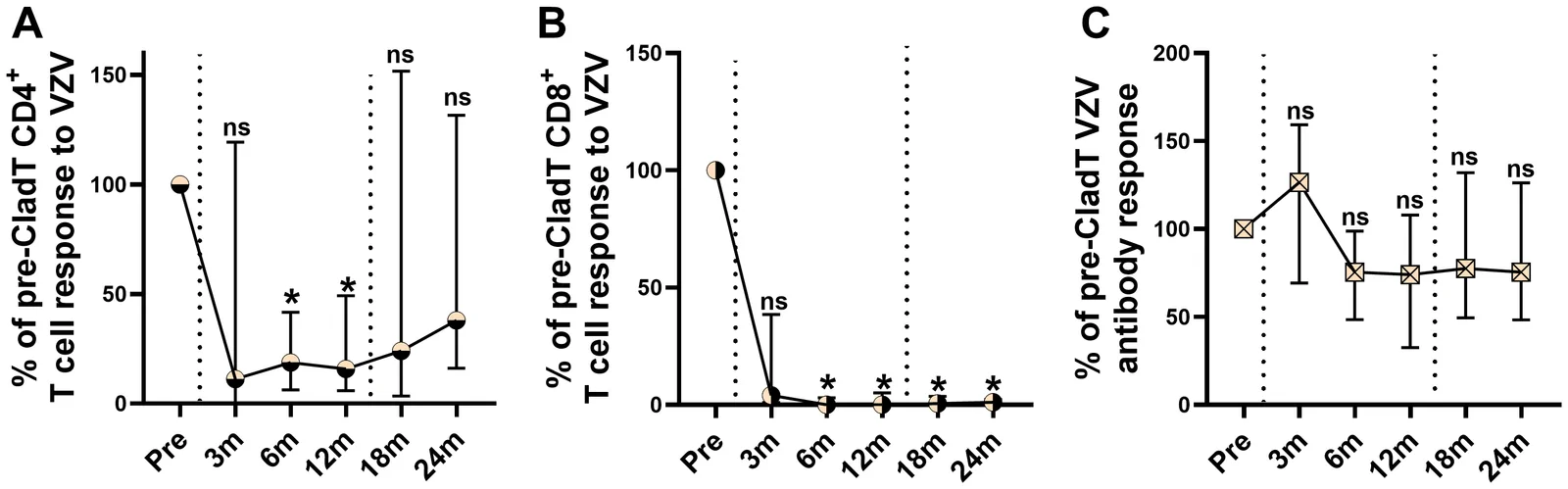
Changes in levels of VZV-specific T cells and antibodies, compared to the pre-CladT level (Pre – set at 100%), at the various timepoints. Each symbol represents the median response and the error bars show the interquartile range of responses. **(A)** CD4^+^ T cell responses (n=9). **(B)** CD8^+^ T cell responses (n=4). **(C)** Anti VZV antibody levels (n=18). The vertical dotted lines represent the two treatments with CladT. Statistical analysis: Friedman test with Dunn’s correction for multiple comparisons. Compared to pre-CladT timepoint, *P<0.05; ns, not significant.

Interestingly, similar to what was observed with the T cell responses, VZV antibody levels increased in 11/18 subjects at the 3-month timepoint and in 7/18 subjects at the 18-month timepoint, immediately after the first or second CladT treatment, respectively (**Figure 6C**).

#### 3.2.3 Tetanus-specific T cell and antibody responses

None of the 5 people in the study who showed T cell responses specific for tetanus toxoid at the pre-CladT timepoint were revaccinated during the study. After the second dose of CladT, there were almost no tetanus toxoid-specific T cells remaining in any patients (**Figure 7A**). Despite few T cells responses being detected, all individuals in the study had measurable levels of antibodies against tetanus toxoid, which showed no significant changes over time at the population level (**Figure 7B**), although the range of antibody concentrations varied from one person to another (**Figure 7C**), and in some individuals who initially had very high levels of antibody, they dropped off markedly over the course of the study (e.g. CLAD10, whose tetanus toxoid-specific antibody levels decreased from 13.5 IU/mL to 1.6 IU/mL). The assay used to detect tetanus toxoid antibody levels was calibrated to determine if the antibody levels are likely to be protective. Two patients had levels below the protective level for antibodies against tetanus at one or more timepoints during the study (**Figure 7C**).

**Figure 7:**
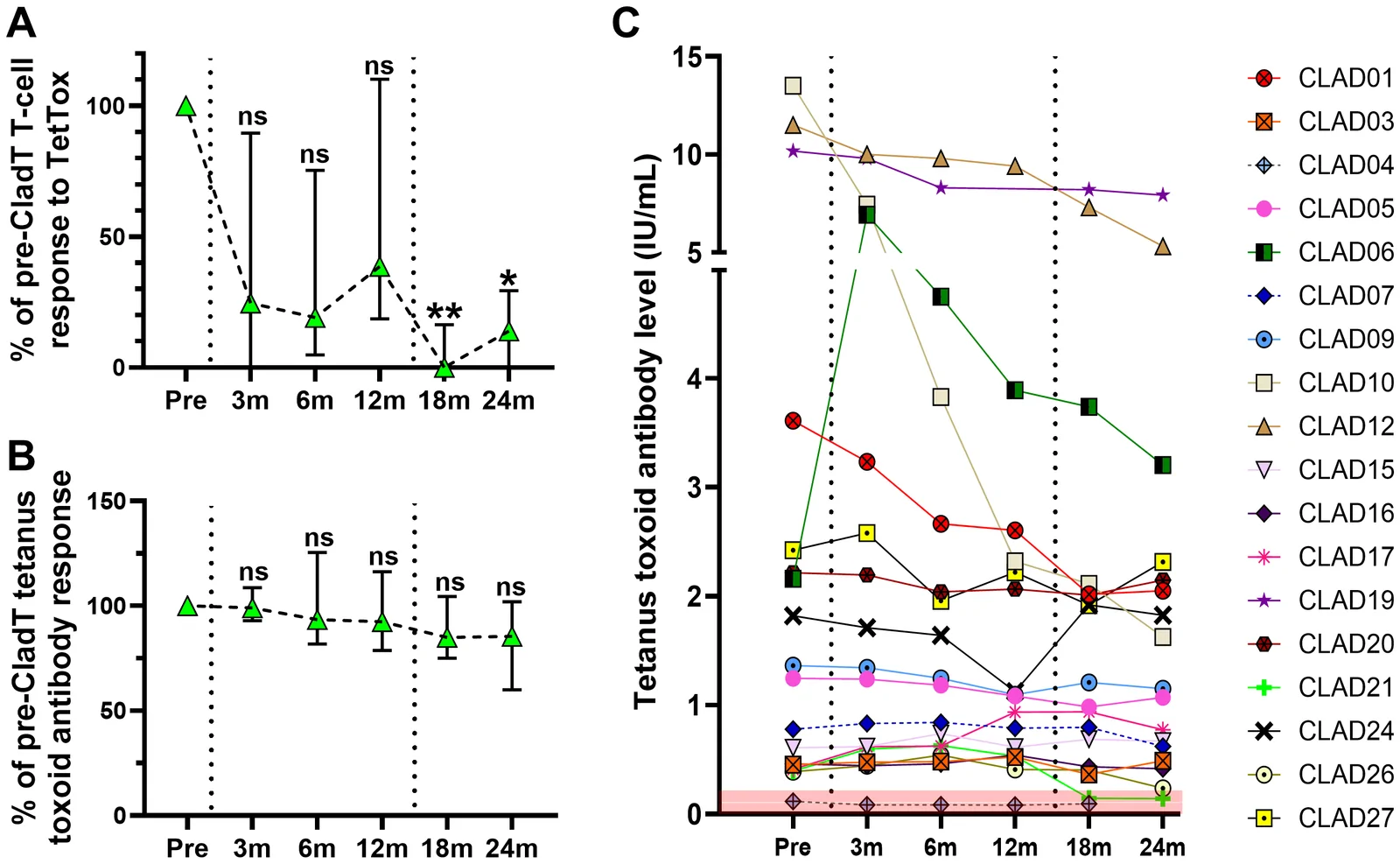
Effects of CladT treatment on levels of tetanus toxoid-specific T cells and antibodies. **(A)** CD4^+^ T cell responses (n=5), shown as a percentage of the pre-CladT level, at the various timepoints. Each symbol represents the median response and the error bars show the interquartile range of responses. **(B)** Overview of median anti-VZV antibody levels (n=18). **(C)** The tetanus toxoid-specific antibody levels (in IU/mL) for each patient. The red shading at the bottom of the graph indicates the cut-off for protective levels of antibodies (0.15 IU/mL). In all figures, the vertical dotted lines represent the two treatments with CladT. Statistical analysis **(A, B)** Friedman test with Dunn’s correction for multiple comparisons. Compared to pre-CladT timepoint, **P<0.01, *P<0.05; ns, not significant.

#### 3.2.4 SARS-CoV-2-RBD-specific antibody responses

At the population level, levels of SARS-CoV-2-specific antibodies were maintained during the first year, but significant decreased after the second course of CladT treatment (**Figure 8**). Nine people were vaccinated against SARS-CoV-2 during the first year following CladT therapy, but only 2 received a vaccination following the second CladT treatment. The large error bars reflect the variations in the number and timing of vaccinations against these antigens amongst the group. Two individuals developed Covid-19 within 3 months of the first CladT treatment, and one developed the disease after the second CladT treatment (**Table 1**).

**Figure 8:**
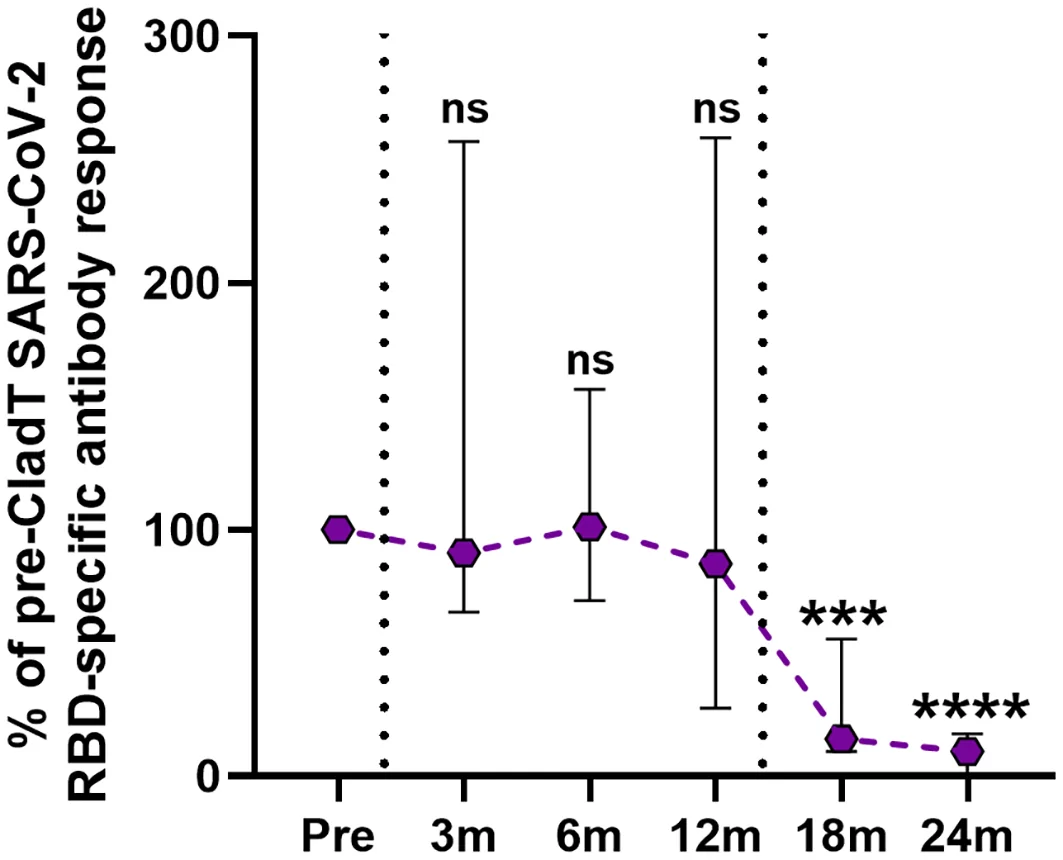
Overview of the median anti-SARS-CoV-2 RBD antibody levels in patients (n=18), compared to the pre-CladT level (Pre – set at 100%), at the various timepoints. The vertical dotted lines represent the two treatments with CladT. Statistical analysis: Friedman test with Dunn’s correction for multiple comparisons. Compared to pre-CladT timepoint, ****P<0.0001, ***P<0.001; ns, not significant.

### 3.3 Effects of prior disease-modifying therapies on immune cell numbers

Fourteen people in the study had been on several other disease-modifying therapies (DMTs) prior to changing to CladT (**Table 1**). Some of the prior DMTs used, specifically alemtuzumab, teriflunomide and dimethyl fumarate, are known to have long-lasting immunological impacts following treatment cessation. Alemtuzumab, which depletes CD52^+^ lymphocytes, NK cells, and myeloid cells, has been reported to lead to long term (> 2 years) depletion of CD8^+^ and CD4^+^ lymphocytes (16), but also increases in the percentages of regulatory T cells (17). Teriflunomide is a cytostatic drug that limits immune cell replication; it has a very long half-life, due to enterohepatic recirculation, and can take at least 2 years to naturally clear from the body. Dimethyl fumarate has been reported to have long-term impacts on lymphocyte biology, with sustained reductions in CD8^+^ T cells (18, 19). The baseline numbers of lymphocyte subsets in individuals who had been on one of these DMTs with long-term biological effects (n=7) were compared with those of people who had no prior DMT exposure (n=4) or those on DMTs that are more rapidly cleared from the body (copaxone and natalizumab; n=7) (**Supplementary Figure S4**). With these small sized groups, there were no significant differences between the groups, although, the number of CD8^+^ T cells/million total lymphocytes was approaching significance, with lower numbers in patients treated with alemtuzumab, teriflunomide, or dimethyl fumarate. To assess whether prior DMTs affected the percent changes in leukocytes and more specifically lymphocyte subgroups, compared to pre-cladribine levels, the data described in **Figures 1** and **2** was reanalyzed, split by prior DMT use (**Supplementary Figures S5A, B**). Again, the small group sizes were underpowered to detect small differences, but one comparison that approached significance was that, contrary to expectations, the majority of central and effector memory T cells that were increased after CladT therapy were present in patients treated with alemtuzumab, teriflunomide, or dimethyl fumarate. There were no clear correlations between antigen-specific vaccine responses and prior use of any particular DMT, with the possible exception of CLAD20, who had stopped treatment with teriflunomide 6 weeks prior to commencing CladT; they were the only participant who did not have detectable levels of T cells specific for influenza and their influenza-specific antibody levels were very low.

## 4 Discussion

In this study we investigated the effects of CladT therapy on numbers of T cell and B cell subsets and on responses to antigens against which patients had previously been vaccinated over a period of 2 years. B cells are important in MS pathogenesis, and many studies of the effects of CladT in MS have focused on B cells and antibodies, and it has been shown in several studies that there is a sustained reduction in B cells, particularly memory B cells, following CladT therapy (7–9, 11, 20, 21). Our study similarly showed sustained reductions in these cells (**Figure 3**). There was a dichotomous response amongst patients for effects of CladT on numbers of CD138^+^ B cells in the blood, which were preserved or even increased in some patients but decreased to low levels in others. These CD138^+^ cells are most likely short-lived antibody-secreting plasmablasts, rather than long-lived plasma cells (which are only present in the circulation in very small numbers), although there are reports in other autoimmune diseases that, following immunodepletion, plasma cells can emerge from the bone marrow and move into the periphery (22). A significant proportion of plasma cells maintain long-term (>1 year) antibody secretion independently of detectable memory B cells (23) and are relatively resistant to cladribine (20). Over the two-year timeline of the study, pre-existing levels of antibodies specific for influenza, tetanus toxoid and VZV were not significantly altered by CladT treatment, which would be consistent with plasma cell survival. Additional markers [eg IRF4, BLIMP-1 and XBP1 (24)] could be used in future studies to resolve the nature of these CD138^+^ cells.

T cells are also critically important in MS pathogenesis. Increased levels of circulating CD4^+^ and CD8^+^ T cells reactive with myelin antigens have been associated with MS (25–29). CD8^+^ T_EMRA_ cells [terminally differentiated, senescent cells (30)] have been associated with progressive MS (31). In addition, T cell help is generally required for optimal high-affinity class-switched antibody responses to form. However, effects of CladT on T cells (other than at a very broad level) have only infrequently been reported (8, 32).

In the current study, subdividing the T cells into CD4^+^ and CD8^+^ subsets and by their memory phenotype showed that, while naïve T cells and T_EMRA_ were chronically depleted by CladT treatment, T_CM_ and T_EM_ typically did not show such levels of depletion, although after the second course of CladT administration at the end of the first year, levels of CD4^+^ T_EM_ did drop to levels similar to the naïve CD4^+^ T cells. Unexpectedly, CD8^+^ T_CM_ and T_EM_ from around one-third of patients markedly increased in number, rather than decreasing, following treatment with CladT. The reasons for this are currently being further investigated; however, we hypothesize that it may be due to elevated CD8^+^ T cell responses to latent herpesviruses (e.g. cytomegalovirus, VZV, Epstein-Barr virus) that are reactivated following CladT treatment, similar to what is frequently seen in immunocompromised patients (33). Surprisingly, patients who had been treated with prior DMTs that have been reported to have long-lasting depleting effects on CD8^+^ T cells (alemtuzumab and dimethyl fumarate) had some of the highest levels of CD8^+^ T_CM_ and T_EM_, suggesting that there are likely to be multiple factors that affect the overall numbers of these cells.

Memory T cells play an important role, both in protective humoral immune responses to vaccine antigens and for rapid cytotoxic cell responses following a secondary encounter with a virus or bacterium. For example, it has been found that the strength of VZV-specific T cell-mediated immunity, but not humoral immunity, that develops after vaccination correlates inversely with subsequent development of herpes zoster (34). Similarly, the T cell response is important in control of influenza (35), with T cells providing protection independently of antibody (36). There is also a T cell response to tetanus toxoid vaccination (37), although the protective mechanism against tetanus primarily involves antibody-mediated inactivation of the tetanus toxin. In our study, we investigated vaccine antigen-specific immune responses after CladT therapy and found that, for influenza, VZV, and tetanus toxoid, memory T cell responses generally decreased over time (**Figures 4**, **6**, **7**; **Supplementary Figure 2**), which could have clinical consequences if patients are not revaccinated to boost surviving T cells or induce new vaccine-antigen specific immunity. For the influenza-specific and VZV-specific CD4^+^ T cells, this decrease was not just due to the overall level of depletion of the memory T cells (**Figure 4C**; **Supplementary Figure S3**). Numbers of individuals with detectable levels of VZV-specific CD8^+^ T cells or tetanus-toxoid-specific CD4^+^ T cells were insufficient for this type on analysiss to be done on these subsets of cells.

Seventeen of the 18 people in the study had pre-existing influenza-specific T cells in their blood - the only patient who did not have any pre-existing T cell reactivity to influenza (CLAD20) had not had any vaccinations, (apart from SARS-CoV-2) since childhood and had only recently discontinued treatment with teriflunomide. Of the 17 individuals with influenza-specific T cells, 13 were vaccinated at least once during the study. All 13 were vaccinated during the 6–12 months following the first CladT treatment (prior to the second dose of cladribine), but 6 of them received another vaccination during months 18-24. There was an elevation in the number of influenza-specific T cells following vaccination at each of those timepoints; however, in the patients who only received the vaccination at 6–12 months, the numbers of influenza specific T cells rapidly decreased following the second CladT treatment, so that those individuals who did not receive the second vaccination had numbers of influenza-specific T cells similar to those who did not receive any vaccinations, whereas numbers were significantly higher in the group who received 2 vaccinations (**Figure 4B**). These findings suggest that vaccine-induced cellular recall responses to influenza decline after each CladT treatment, but that revaccination around 18–24 months after treatment initiation, has the potential to restore influenza-specific responses. We do not know whether the responses after revaccination are due to expansion of the same clones as were present before CladT treatment or due to newly arising clones; this would be a subject for future research.

The other antigen against which most patients were vaccinated during the course of this study was SARS-CoV-2. There could be several reasons for the significant decline in antibody levels at the last two timepoints (**Figure 8**). It could be that the effect of the second dose of CladT was sufficient to deplete that response, as was seen was seen with influenza, especially given that only 2 individuals were vaccinated against SARS-CoV-2 during the last half of the study. However, another possibility is that this drop-off in antibody levels was due to the timing of blood collections in relation to vaccine roll-out in Queensland, Australia, where this study was undertaken, during the Covid pandemic. Most workplaces mandated a minimum of 2 vaccinations in order for people to be able to access their workplace. Overall, numbers of cases of Covid-19 were very low in Queensland during the period of the study, and therefore, after those two vaccinations, many people elected not to have further vaccinations. The durability of the responses to the earliest SARS-CoV-2 vaccines was not optimal, and most people required several vaccinations and/or infections to build up a strong immune response.

Although there have been concerns about the possibility of MS relapse after vaccination, we found no adverse effects of vaccination, as has also been the case with other studies in MS (38, 39). Vaccination in MS, including following CladT therapy, has been found to be safe and effective in most people (including pediatric patients) with MS (15, 40). We suggest that clinicians need to be aware of the need for revaccination after immune therapy. Indeed, this is not only the case for MS but has been highlighted as a widespread problem in immune mediated disease (41).

Interestingly, 5/9 patients who showed detectable levels of VZV-specific CD4^+^ T cells showed transient increases in numbers of these cells within the period just after CladT therapy. This could be compatible with transient expansion of the VZV-specific T cells to deal with a subclinical reactivation of VZV, or it could be due to bystander activation or homeostatic proliferation. Future studies should include viral PCR, serological markers and more thorough clinical surveillance to determine exactly what the case is.

## 5 Summary and limitations of the study

In summary, we have further expanded knowledge of the changes in T cells and B cells after CladT. We confirm that CladT treatment is associated with broad lymphocyte depletion, a sustained reduction in numbers of memory B cells and variable effects on T cell subsets. Pre-existing levels of antibodies specific for influenza, tetanus toxoid and VZV were not significantly affected by CladT treatment over the two year timeline of the study. However, there was a decline in detectable antigen-specific memory T cells over time, especially after the second course of treatment. This is important, particularly for vaccines where protection against infection relies on the presence of both antibodies and T cells specific for the infective agent (e.g. influenza and VZV vaccines). For influenza at least, antigen-specific memory T cells could be restored to pre-treatment levels by revaccination after the second treatment course. Larger studies are needed to define antigen-specific revaccination schedules more broadly.

The main limitation of the study is the relatively small cohort size and the real-world nature of the study (i.e. patients vaccinated at different times with different vaccine formulations and treated with a variety of DMTs prior to CladT). This limits the statistical power and generalizability of the findings. especially for data where patients were grouped by prior DMTs, and in the VZV and tetanus toxoid T cell assays, where numbers of patients with detectable levels of antigen-specific T cells pre-CladT therapy were lower. Data in these areas therefore remain exploratory and hypothesis generating.

## Data Availability

The original contributions presented in the study are included in the article and **Supplementary Material**. Further inquiries can be directed to the corresponding author.
