# Supplementary material for: Longitudinal analysis of the effects of cladribine tablets on B and T cell memory recall responses to vaccine antigens in people with multiple sclerosis
Source: Front Immunol. 2026 Oct 22;17:1881334. doi: 10.3389/fimmu.2026.1881334 (PMC13735715; doi:10.3389/fimmu.2026.1881334)
Supplement: Supplementary file 1 [file DataSheet1.pdf]

Supplementary Material

Longitudinal analysis of the effects of cladribine tablets on B and T cell memory recall responses to vaccine antigens in people with multiple sclerosis

Peter A Csurhes, Pamela A McCombe, Judith M. Greer*

Frazer Institute

The University of Queensland,
Brisbane, Queensland, Australia

1 Supplementary Tables

1.1 Supplementary Table S1. Flow cytometry antibody panels used in this study.

A. Whole blood count cytometry panel

Channel	Marker	Fluorochrome
FL 1	CD3	FITC
FL 2	CD19	PE
FL 3	CD16	PE-CF594
FL 3	CD56	PE-CF594
FL 4	CD4	PerCP Cy5.5
FL 5	CD27	PE-Cy7
FL 6	CD14	APC
FL 7	CD138	APC-R700
FL 8	CD8	APC-Cy7
FL 9	CD45	BV421

B. Intracellular cytokine cytometry panel

Channel	Marker	Fluorochrome
FL 1	IFN	FITC
FL 2	CD4	PE
FL 3	CD8	PE-CF594
FL 5	CD45RA	PE-Cy7
FL 6	IL-2	APC
FL 7		
FL 8	CD62L	APC-Cy7
FL 9	TNFa	BV421
FL 10	Aqua viability dye	

2 Supplementary Figures

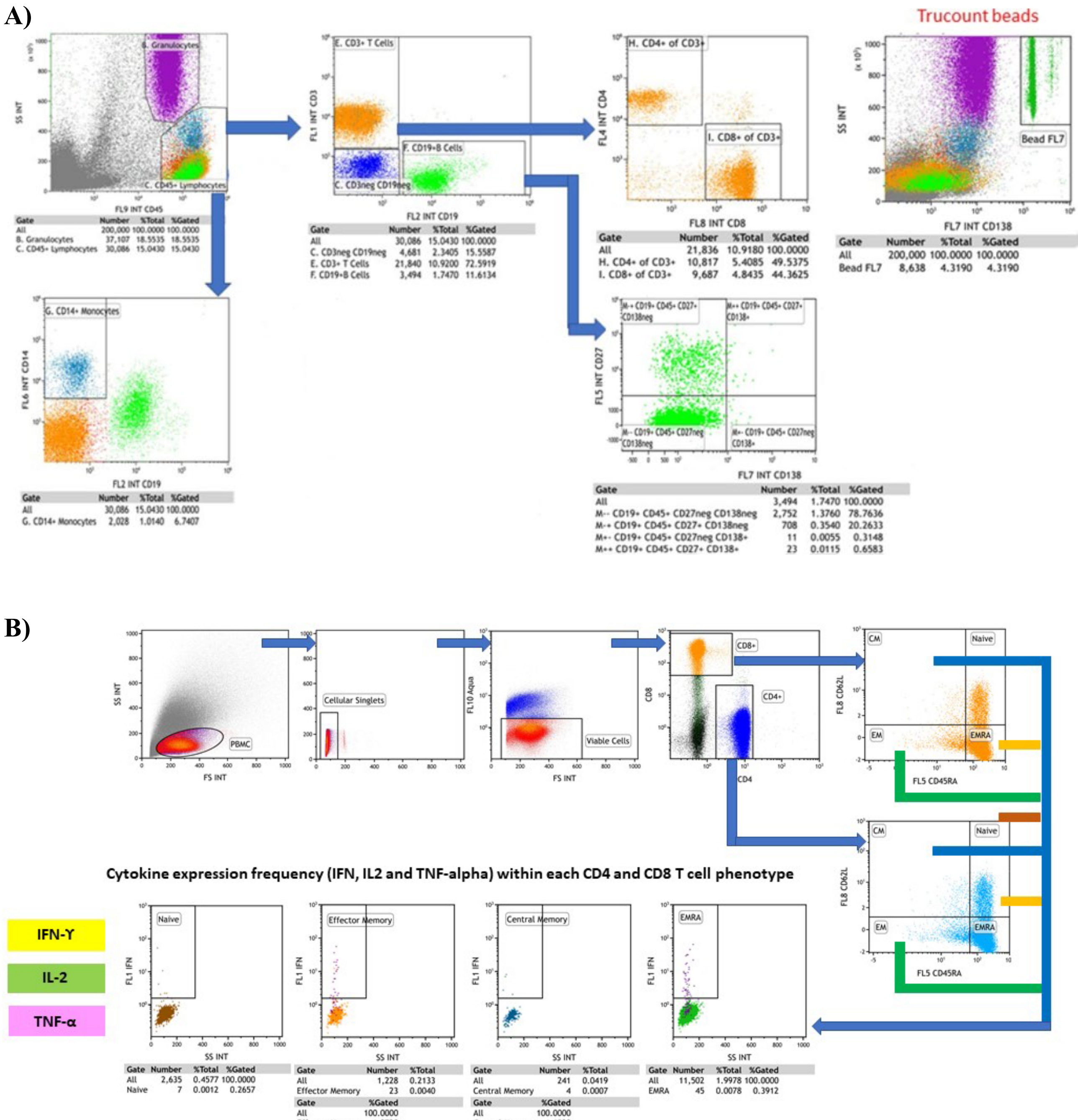

Supplementary Figure S1. Examples of flow cytometry gating strategies for: A) using Trucount beads to enumerate numbers of the various cell subsets investigated in this study; and B) intracellular cytokine staining to measure the frequency of cytokine-producing vaccine-specific T cells within each T cell memory subset. Single-labelled tubes for each antibody, isotype-matched control antibodies, fluorescence-minus-one controls, dead cell exclusion dyes and doublet discrimination were used extensively during panel development to ensure accurate positive cutoff values and compensation matrices and to validate cell phenotype detection sensitivity and resolution.

Supplementary Figure S2 i) – xviii). (pages 5-22)

Vaccine antigen-specific T cell and antibody responses and B and T cell numbers in individual patients. Interpretation of each component in this figure is as follows:

The ID number (CLAD01, 03 etc) and demographic details (age at commencement of cladribine treatment and sex) of the individual tested is shown at the top of each set of 5 graphs. In all graphs, the vertical dotted line at Day 0 represents the first day of administration of cladribine. The next dotted vertical line represents the day of starting the second dose of cladribine. Arrows below the baseline in panel A and B indicates the day of vaccination against influenza (red arrow), VZV (beige arrow), tetanus toxoid (green arrow) or Covid (purple arrow). Only vaccinations done in the 3 months prior to the first cladribine course are shown before day 0 on the x axis.

Panel A shows the T cell responses to vaccine antigens (influenza, VZV, and tetanus toxoid). The number of IFNγ and/or TNF-producing T cells/mL specific for each antigen at the pre-administration timepoint is shown under each antigen in the figure legend and is set to 100%, except when the number of cells detectable did not meet the criteria for a positive response. Red circles joined by a dashed line show the response to influenza, VZV $CD4^+$ responses are indicated by beige coloured squares with a cross inside joined by a solid black line, VZV CD8+ responses are indicated by a square (half beige and half black) and tetanus toxoid (Tet tox) responses are indicated by green triangles joined by a black dashed line.

Panel B shows the antibody response to the same antigens as in A, with the inclusion also of SARS-CoV-2. Antibody responses to SARS-CoV-2 are shown by purple hexagons connected by purple dashed lines.

Panel C shows the levels of $CD19^+$ B cells and $CD3^+$ T cells in patient blood at each timepoint (the pre-administration timepoint is set as 100%, and the number of cells at that timepoint is shown in the figure legend), and panels D and E further split the T cells into $CD4^+$ or $CD8^+$ naïve, T_{CM}, T_{EM} and T_{EMRA} in patient blood at each timepoint (pre-administration timepoint is set as 100% level). The legends to the right of each panel describe the symbols used for each of the different cell populations.

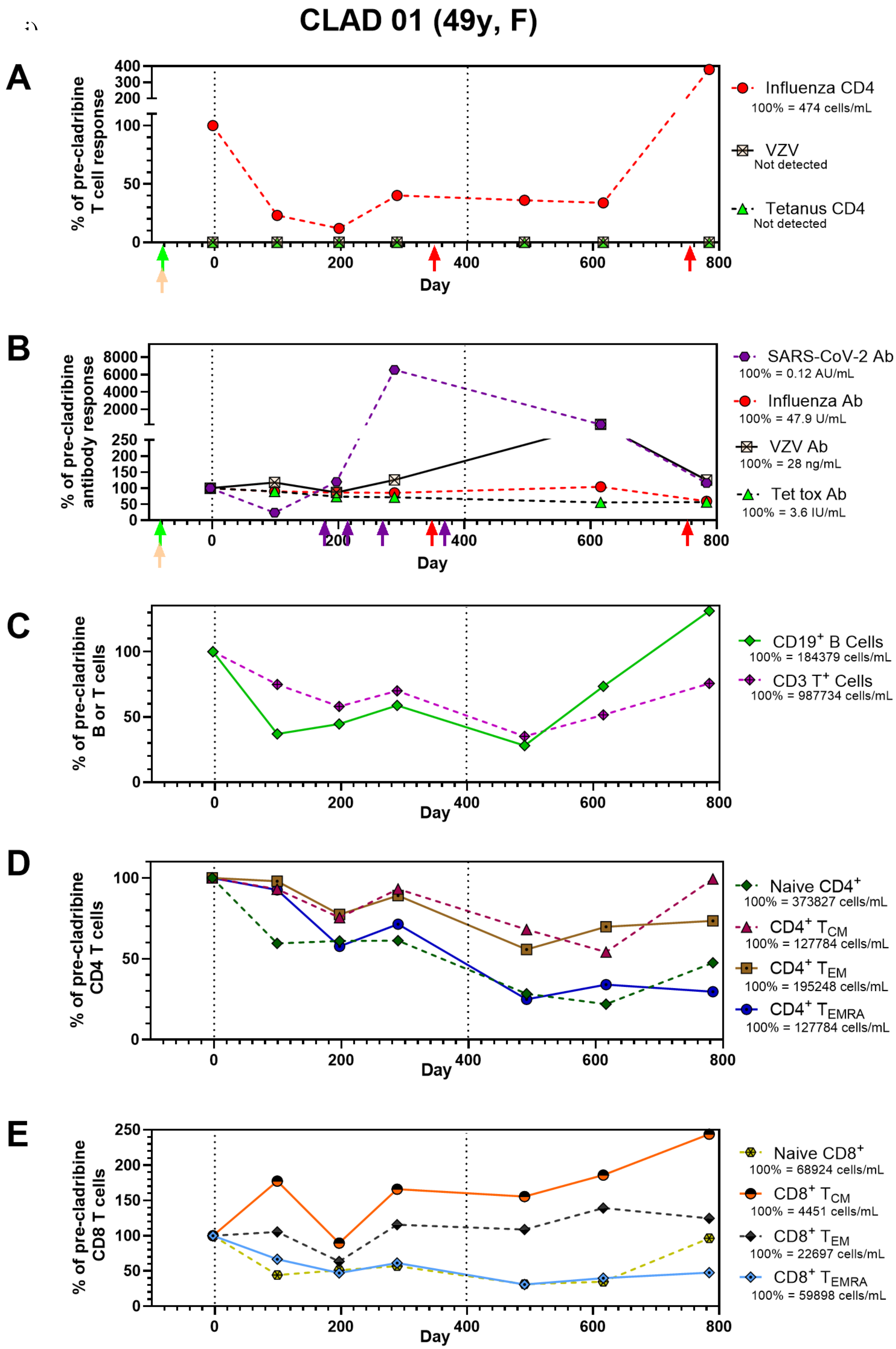

CLAD 01 (49y, F)
A
% of pre-cladribine T cell response
Influenza CD4
100% = 474 cells/mL
VZV
Not detected
Tetanus CD4
Not detected
Day
B
% of pre-cladribine antibody response
SARS-CoV-2 Ab
100% = 0.12 AU/mL
Influenza Ab
100% = 47.9 U/mL
VZV Ab
100% = 28 ng/mL
Tet tox Ab
100% = 3.6 IU/mL
Day
C
% of pre-cladribine B or T cells
CD19+ B Cells
100% = 184379 cells/mL
CD3 T+ Cells
100% = 987734 cells/mL
Day
D
% of pre-cladribine CD4 T cells
Naive CD4+
100% = 373827 cells/mL
CD4+ TCM
100% = 127784 cells/mL
CD4+ TEM
100% = 195248 cells/mL
CD4+ TEMRA
100% = 127784 cells/mL
Day
E
% of pre-cladribine CD8 T cells
Naive CD8+
100% = 68924 cells/mL
CD8+ TCM
100% = 4451 cells/mL
CD8+ TEM
100% = 22697 cells/mL
CD8+ TEMRA
100% = 59898 cells/mL
Day

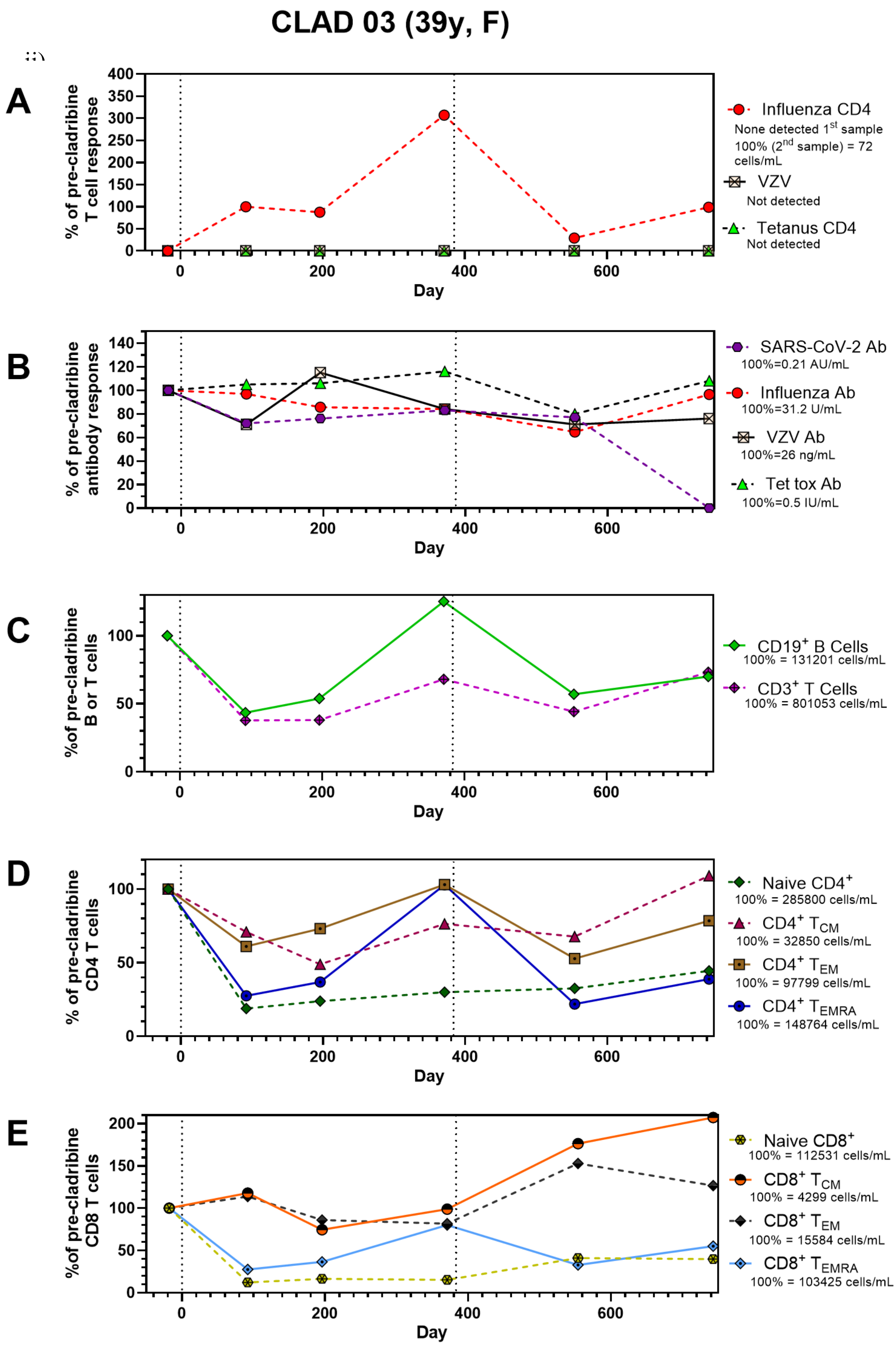

CLAD 03 (39y, F)
A
% of pre-cladribine T cell response
Day
Influenza CD4
None detected 1st sample
100% (2nd sample) = 72 cells/mL
VZV
Not detected
Tetanus CD4
Not detected
B
% of pre-cladribine antibody response
Day
SARS-CoV-2 Ab
100%=0.21 AU/mL
Influenza Ab
100%=31.2 U/mL
VZV Ab
100%=26 ng/mL
Tet tox Ab
100%=0.5 IU/mL
C
%of pre-cladribine B or T cells
Day
CD19+ B Cells
100% = 131201 cells/mL
CD3+ T Cells
100% = 801053 cells/mL
D
% of pre-cladribine CD4 T cells
Day
Naive CD4+
100% = 285800 cells/mL
CD4+ TCM
100% = 32850 cells/mL
CD4+ TEM
100% = 97799 cells/mL
CD4+ TEMRA
100% = 148764 cells/mL
E
%of pre-cladribine CD8 T cells
Day
Naive CD8+
100% = 112531 cells/mL
CD8+ TCM
100% = 4299 cells/mL
CD8+ TEM
100% = 15584 cells/mL
CD8+ TEMRA
100% = 103425 cells/mL

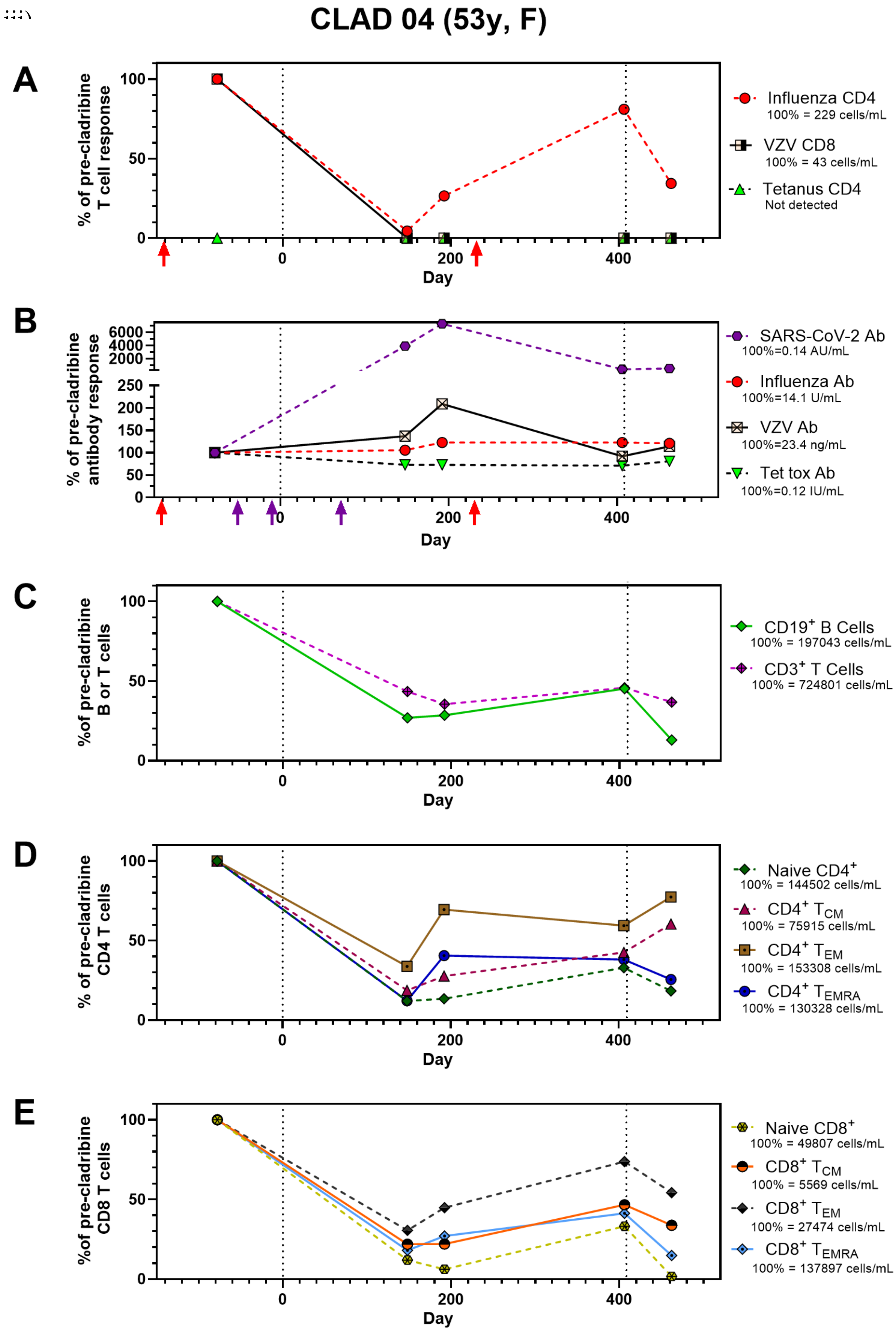

CLAD 04 (53y, F)
A
% of pre-cladribine T cell response
Day
Influenza CD4
100% = 229 cells/mL
VZV CD8
100% = 43 cells/mL
Tetanus CD4
Not detected
B
% of pre-cladribine antibody response
Day
SARS-CoV-2 Ab
100%=0.14 AU/mL
Influenza Ab
100%=14.1 U/mL
VZV Ab
100%=23.4 ng/mL
Tet tox Ab
100%=0.12 IU/mL
C
%of pre-cladribine B or T cells
Day
CD19+ B Cells
100% = 197043 cells/mL
CD3+ T Cells
100% = 724801 cells/mL
D
% of pre-cladribine CD4 T cells
Day
Naive CD4+
100% = 144502 cells/mL
CD4+ TCM
100% = 75915 cells/mL
CD4+ TEM
100% = 153308 cells/mL
CD4+ TEMRA
100% = 130328 cells/mL
E
%of pre-cladribine CD8 T cells
Day
Naive CD8+
100% = 49807 cells/mL
CD8+ TCM
100% = 5569 cells/mL
CD8+ TEM
100% = 27474 cells/mL
CD8+ TEMRA
100% = 137897 cells/mL

CLAD 05 (36y, M)

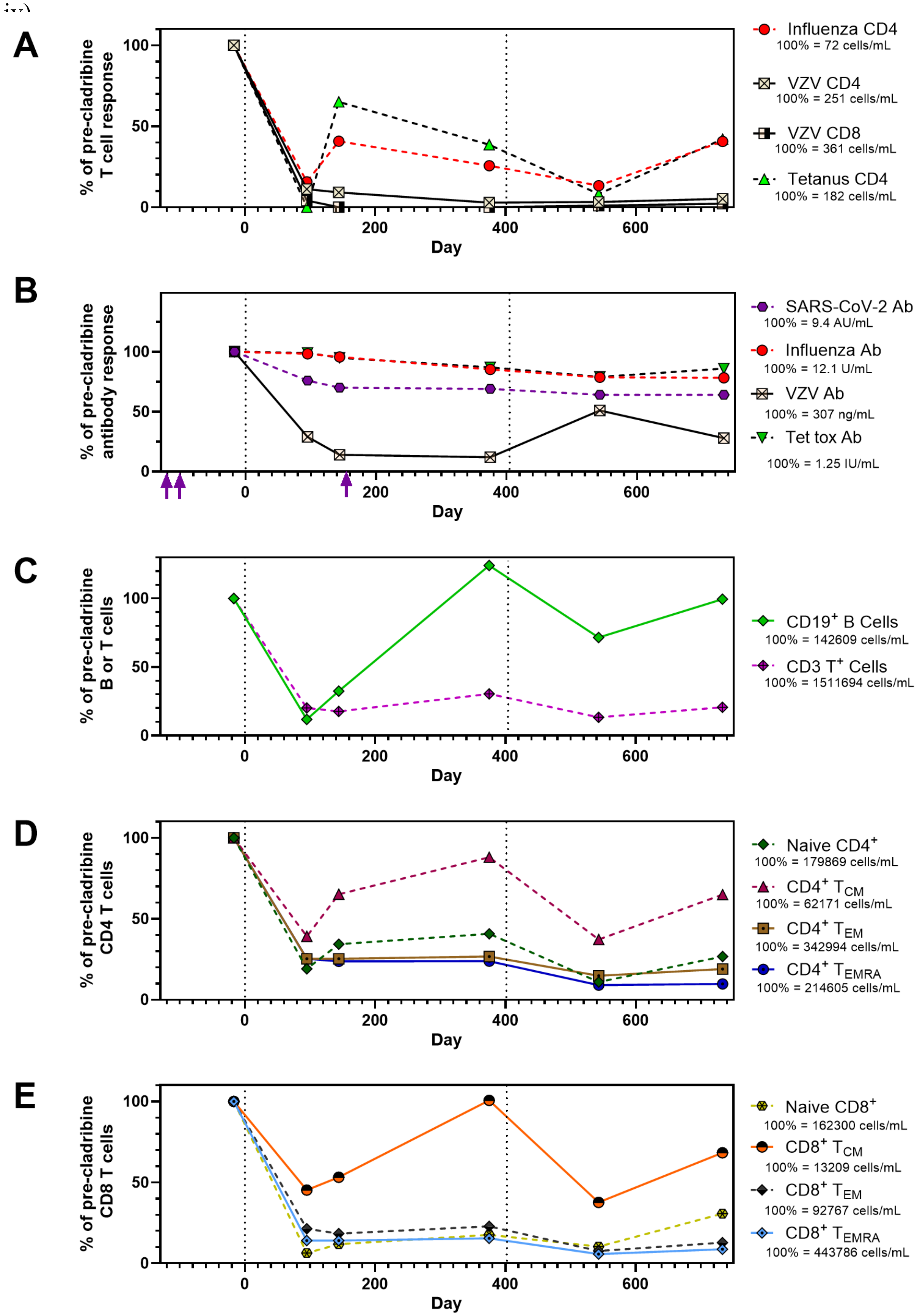
A
% of pre-cladribine T cell response
Day
Influenza CD4
100% = 72 cells/mL
VZV CD4
100% = 251 cells/mL
VZV CD8
100% = 361 cells/mL
Tetanus CD4
100% = 182 cells/mL
B
% of pre-cladribine antibody response
SARS-CoV-2 Ab
100% = 9.4 AU/mL
Influenza Ab
100% = 12.1 U/mL
VZV Ab
100% = 307 ng/mL
Tet tox Ab
100% = 1.25 IU/mL
C
% of pre-cladribine B or T cells
$CD19^+$ B Cells
100% = 142609 cells/mL
CD3 T^+ Cells
100% = 1511694 cells/mL
D
% of pre-cladribine CD4 T cells
Naive $CD4^+$
100% = 179869 cells/mL
$CD4^+$ T_{CM}
100% = 62171 cells/mL
$CD4^+$ T_{EM}
100% = 342994 cells/mL
$CD4^+$ T_{EMRA}
100% = 214605 cells/mL
E
% of pre-cladribine CD8 T cells
Naive $CD8^+$
100% = 162300 cells/mL
$CD8^+$ T_{CM}
100% = 13209 cells/mL
$CD8^+$ T_{EM}
100% = 92767 cells/mL
$CD8^+$ T_{EMRA}
100% = 443786 cells/mL

CLAD 06 (60y, F)

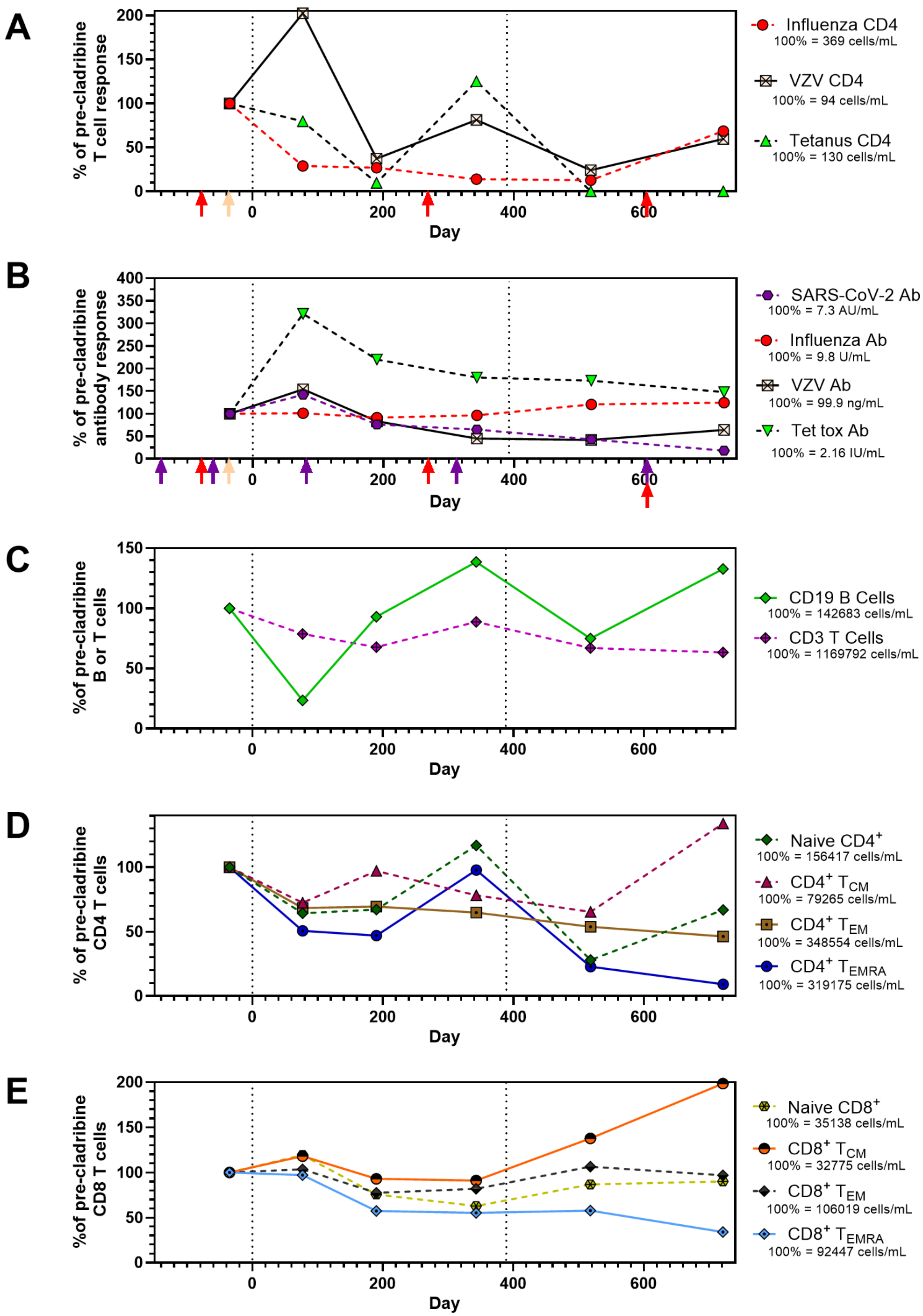
A
% of pre-cladribine T cell response
Day
Influenza CD4
100% = 369 cells/mL
VZV CD4
100% = 94 cells/mL
Tetanus CD4
100% = 130 cells/mL
B
% of pre-cladribine antibody response
Day
SARS-CoV-2 Ab
100% = 7.3 AU/mL
Influenza Ab
100% = 9.8 U/mL
VZV Ab
100% = 99.9 ng/mL
Tet tox Ab
100% = 2.16 IU/mL
C
%of pre-cladribine B or T cells
Day
CD19 B Cells
100% = 142683 cells/mL
CD3 T Cells
100% = 1169792 cells/mL
D
% of pre-cladribine CD4 T cells
Day
Naive CD4+
100% = 156417 cells/mL
CD4+ TCM
100% = 79265 cells/mL
CD4+ TEM
100% = 348554 cells/mL
CD4+ TEMRA
100% = 319175 cells/mL
E
%of pre-cladribine CD8 T cells
Day
Naive CD8+
100% = 35138 cells/mL
CD8+ TCM
100% = 32775 cells/mL
CD8+ TEM
100% = 106019 cells/mL
CD8+ TEMRA
100% = 92447 cells/mL

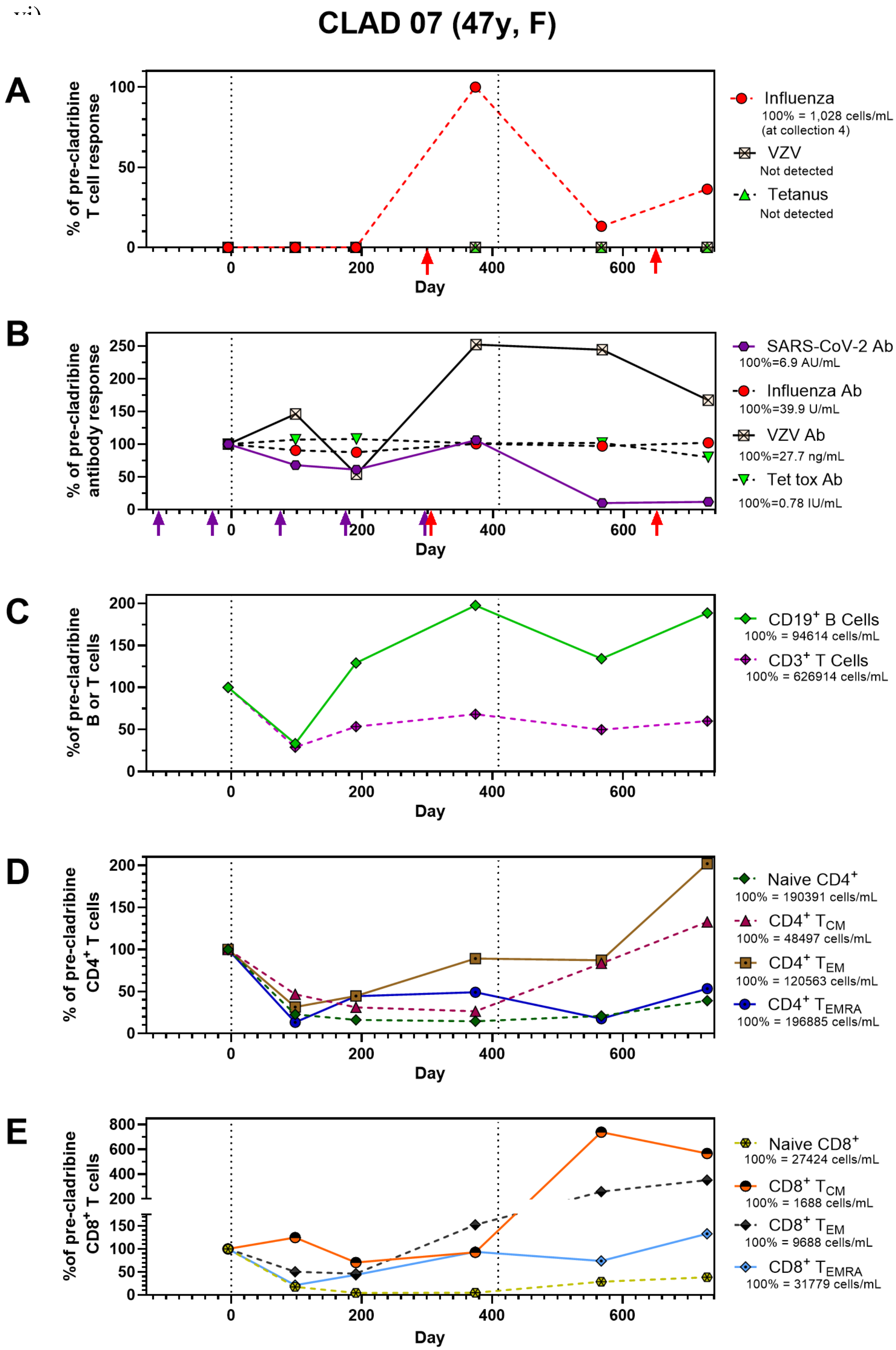
CLAD 07 (47y, F)
A
% of pre-cladribine T cell response
Day
Influenza
100% = 1,028 cells/mL (at collection 4)
VZV
Not detected
Tetanus
Not detected
B
% of pre-cladribine antibody response
Day
SARS-CoV-2 Ab
100%=6.9 AU/mL
Influenza Ab
100%=39.9 U/mL
VZV Ab
100%=27.7 ng/mL
Tet tox Ab
100%=0.78 IU/mL
C
%of pre-cladribine B or T cells
Day
CD19+ B Cells
100% = 94614 cells/mL
CD3+ T Cells
100% = 626914 cells/mL
D
% of pre-cladribine CD4+ T cells
Day
Naive CD4+
100% = 190391 cells/mL
CD4+ TCM
100% = 48497 cells/mL
CD4+ TEM
100% = 120563 cells/mL
CD4+ TEMRA
100% = 196885 cells/mL
E
%of pre-cladribine CD8+ T cells
Day
Naive CD8+
100% = 27424 cells/mL
CD8+ TCM
100% = 1688 cells/mL
CD8+ TEM
100% = 9688 cells/mL
CD8+ TEMRA
100% = 31779 cells/mL

CLAD 09 (36y, F)

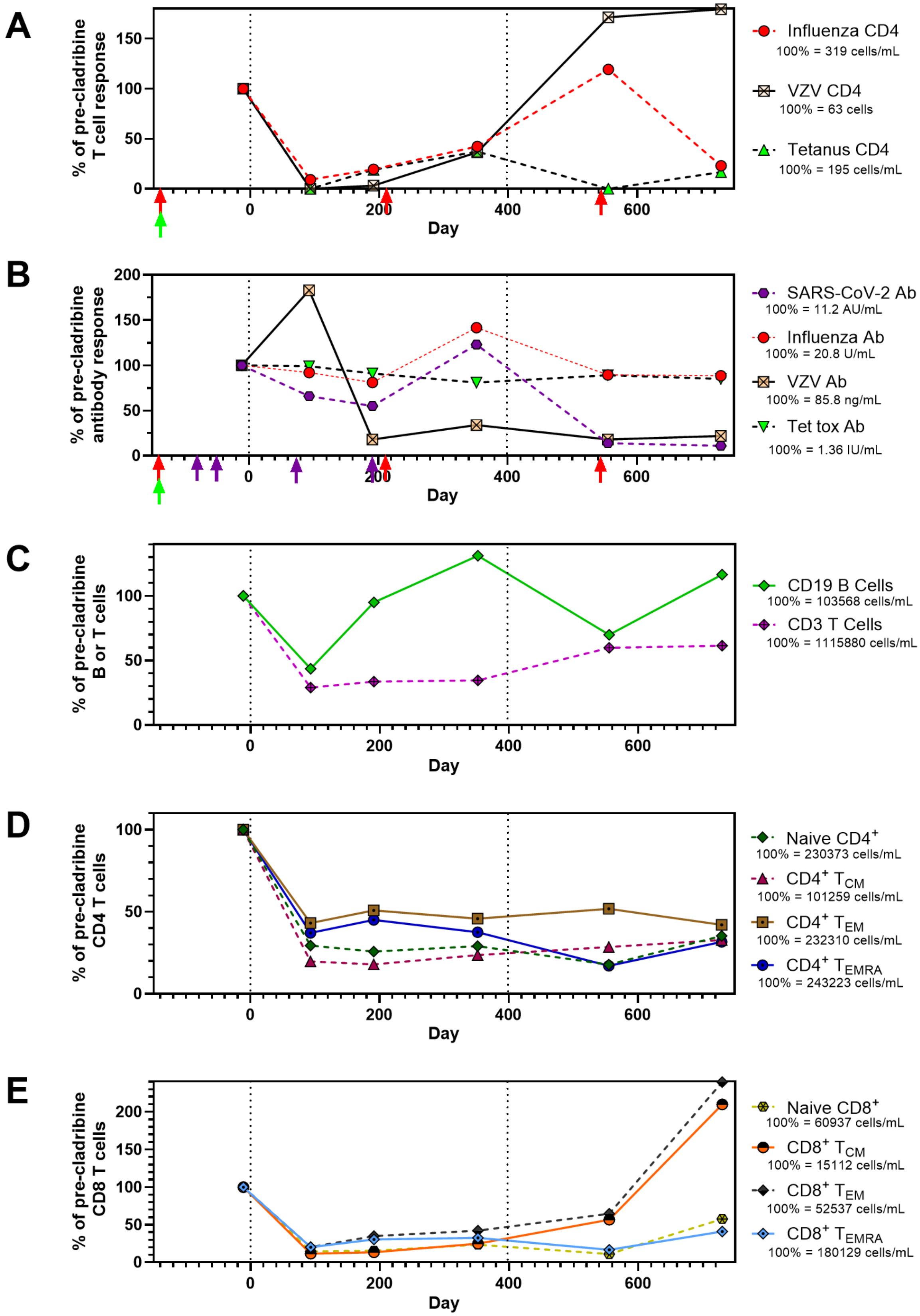
A
% of pre-cladribine T cell response
Day
Influenza CD4
100% = 319 cells/mL
VZV CD4
100% = 63 cells
Tetanus CD4
100% = 195 cells/mL
B
% of pre-cladribine antibody response
Day
SARS-CoV-2 Ab
100% = 11.2 AU/mL
Influenza Ab
100% = 20.8 U/mL
VZV Ab
100% = 85.8 ng/mL
Tet tox Ab
100% = 1.36 IU/mL
C
% of pre-cladribine B or T cells
Day
CD19 B Cells
100% = 103568 cells/mL
CD3 T Cells
100% = 1115880 cells/mL
D
% of pre-cladribine CD4 T cells
Day
Naive $CD4^+$
100% = 230373 cells/mL
$CD4^+$ T_{CM}
100% = 101259 cells/mL
$CD4^+$ T_{EM}
100% = 232310 cells/mL
$CD4^+$ T_{EMRA}
100% = 243223 cells/mL
E
% of pre-cladribine CD8 T cells
Day
Naive $CD8^+$
100% = 60937 cells/mL
$CD8^+$ T_{CM}
100% = 15112 cells/mL
$CD8^+$ T_{EM}
100% = 52537 cells/mL
$CD8^+$ T_{EMRA}
100% = 180129 cells/mL

CLAD 10 (49y, F)

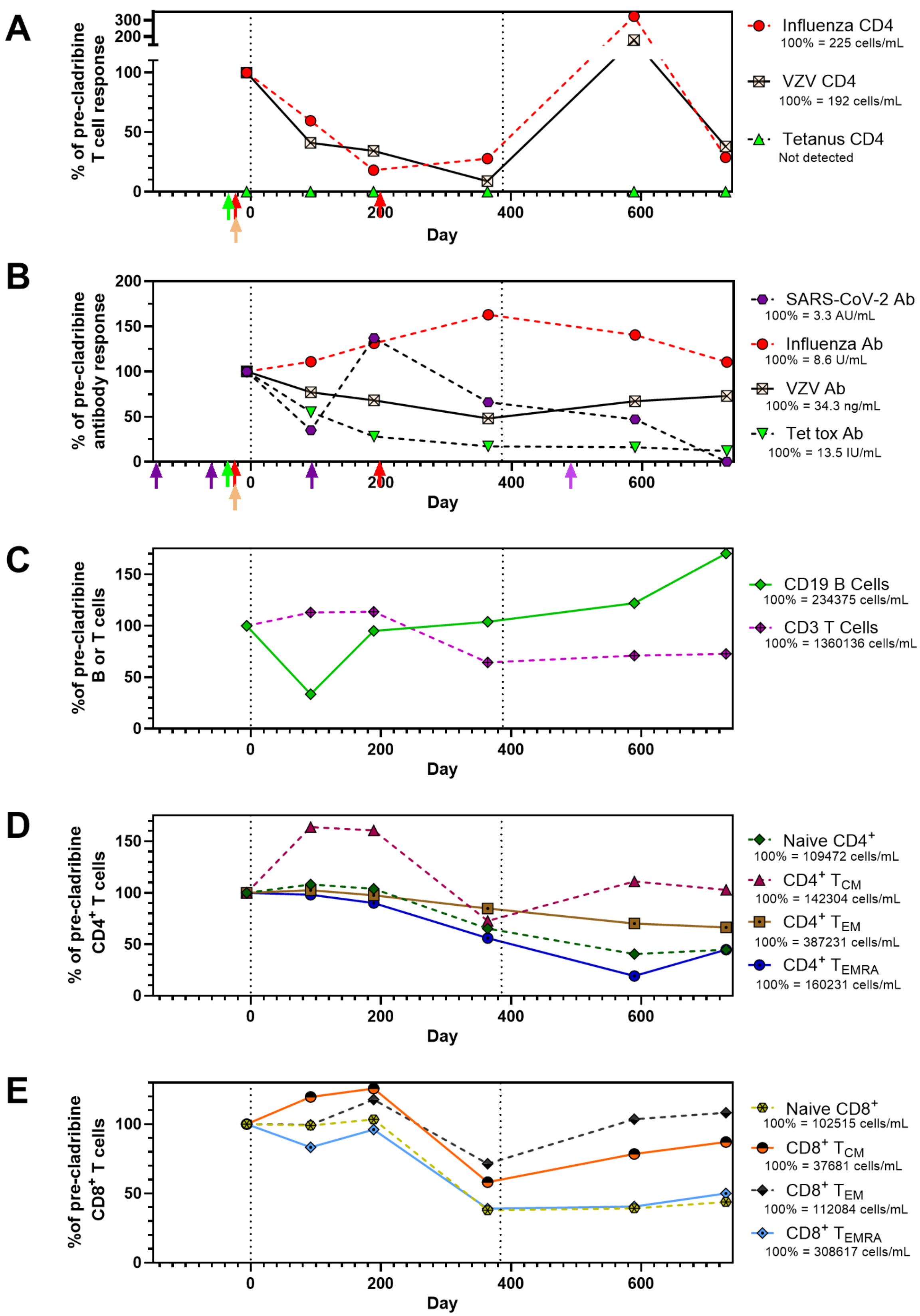
A
% of pre-cladribine T cell response
Day
Influenza CD4
100% = 225 cells/mL
VZV CD4
100% = 192 cells/mL
Tetanus CD4
Not detected
B
% of pre-cladribine antibody response
Day
SARS-CoV-2 Ab
100% = 3.3 AU/mL
Influenza Ab
100% = 8.6 U/mL
VZV Ab
100% = 34.3 ng/mL
Tet tox Ab
100% = 13.5 IU/mL
C
%of pre-cladribine B or T cells
Day
CD19 B Cells
100% = 234375 cells/mL
CD3 T Cells
100% = 1360136 cells/mL
D
% of pre-cladribine CD4+ T cells
Day
Naive CD4+
100% = 109472 cells/mL
CD4+ TCM
100% = 142304 cells/mL
CD4+ TEM
100% = 387231 cells/mL
CD4+ TEMRA
100% = 160231 cells/mL
E
%of pre-cladribine CD8+ T cells
Day
Naive CD8+
100% = 102515 cells/mL
CD8+ TCM
100% = 37681 cells/mL
CD8+ TEM
100% = 112084 cells/mL
CD8+ TEMRA
100% = 308617 cells/mL

CLAD 12 (30y, F)

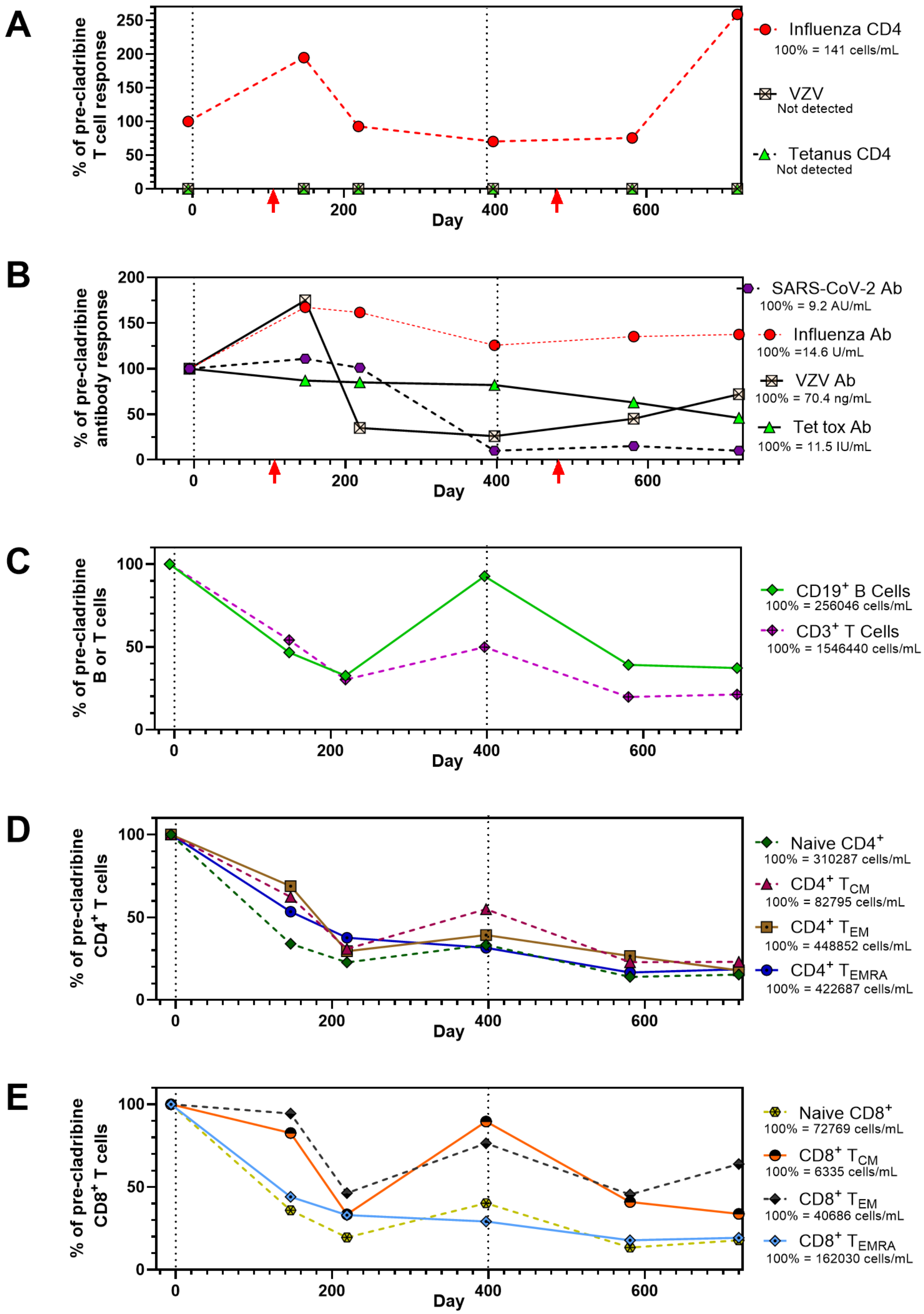

A
% of pre-cladribine T cell response
Day
Influenza CD4
100% = 141 cells/mL
VZV
Not detected
Tetanus CD4
Not detected
B
% of pre-cladribine antibody response
Day
SARS-CoV-2 Ab
100% = 9.2 AU/mL
Influenza Ab
100% =14.6 U/mL
VZV Ab
100% = 70.4 ng/mL
Tet tox Ab
100% = 11.5 IU/mL
C
% of pre-cladribine B or T cells
Day
CD19+ B Cells
100% = 256046 cells/mL
CD3+ T Cells
100% = 1546440 cells/mL
D
% of pre-cladribine CD4+ T cells
Day
Naive CD4+
100% = 310287 cells/mL
CD4+ TCM
100% = 82795 cells/mL
CD4+ TEM
100% = 448852 cells/mL
CD4+ TEMRA
100% = 422687 cells/mL
E
% of pre-cladribine CD8+ T cells
Day
Naive CD8+
100% = 72769 cells/mL
CD8+ TCM
100% = 6335 cells/mL
CD8+ TEM
100% = 40686 cells/mL
CD8+ TEMRA
100% = 162030 cells/mL

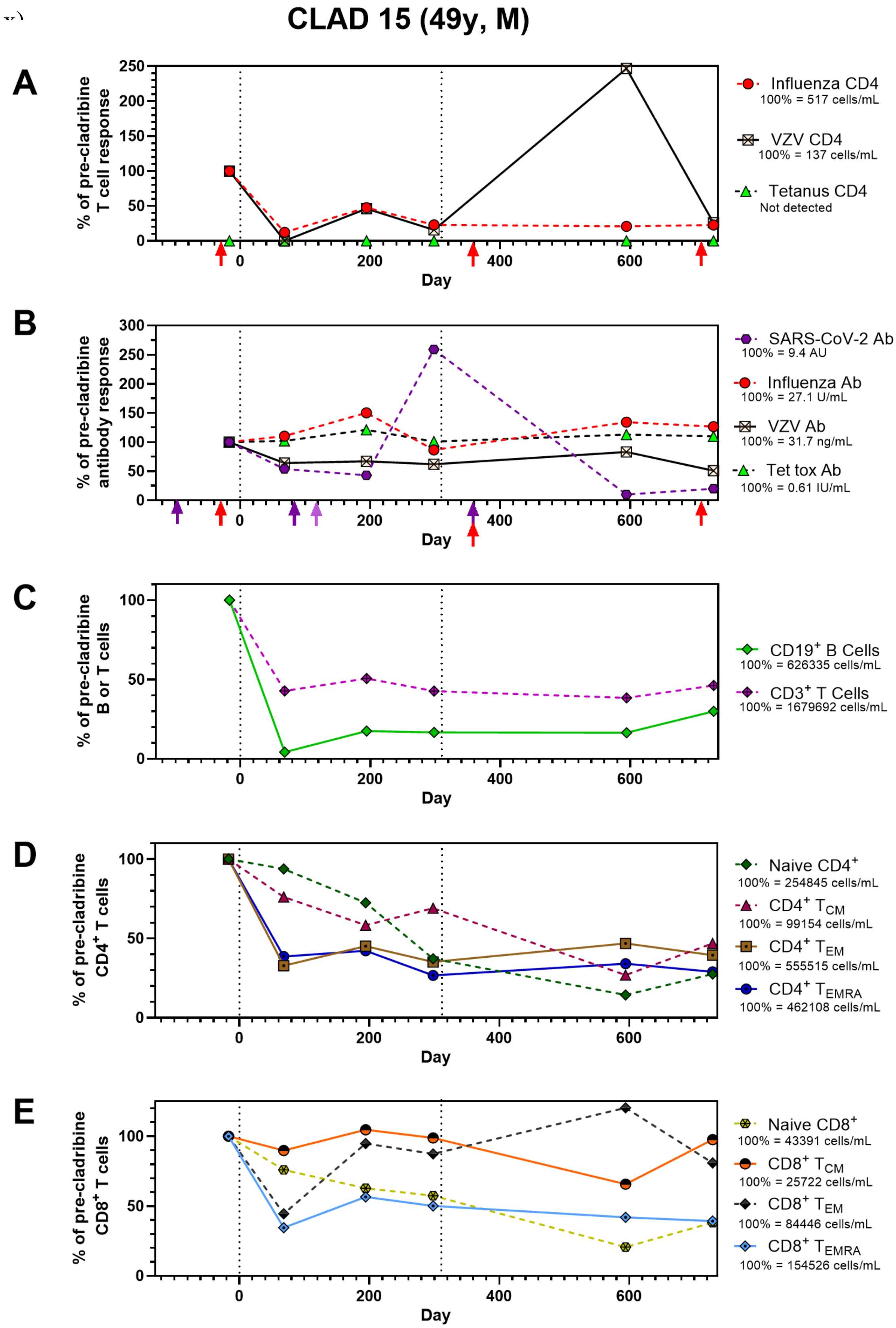
CLAD 15 (49y, M)
A
% of pre-cladribine T cell response
Day
Influenza CD4
100% = 517 cells/mL
VZV CD4
100% = 137 cells/mL
Tetanus CD4
Not detected
B
% of pre-cladribine antibody response
SARS-CoV-2 Ab
100% = 9.4 AU
Influenza Ab
100% = 27.1 U/mL
VZV Ab
100% = 31.7 ng/mL
Tet tox Ab
100% = 0.61 IU/mL
C
% of pre-cladribine B or T cells
CD19+ B Cells
100% = 626335 cells/mL
CD3+ T Cells
100% = 1679692 cells/mL
D
% of pre-cladribine CD4+ T cells
Naive CD4+
100% = 254845 cells/mL
CD4+ TCM
100% = 99154 cells/mL
CD4+ TEM
100% = 555515 cells/mL
CD4+ TEMRA
100% = 462108 cells/mL
E
% of pre-cladribine CD8+ T cells
Naive CD8+
100% = 43391 cells/mL
CD8+ TCM
100% = 25722 cells/mL
CD8+ TEM
100% = 84446 cells/mL
CD8+ TEMRA
100% = 154526 cells/mL

CLAD 16 (41y, F)

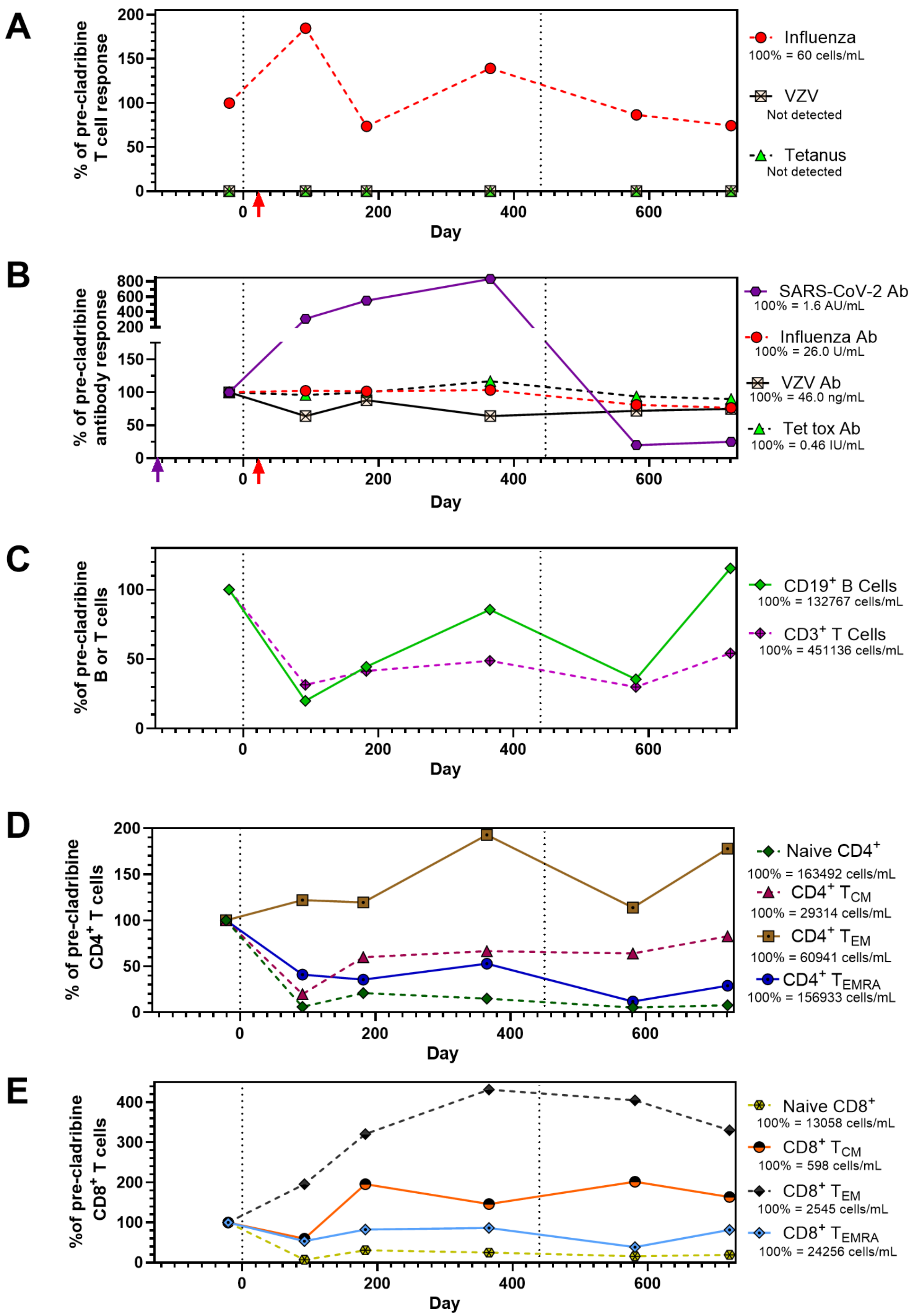
A
% of pre-cladribine T cell response
Day
Influenza
100% = 60 cells/mL
VZV
Not detected
Tetanus
Not detected
B
% of pre-cladribine antibody response
Day
SARS-CoV-2 Ab
100% = 1.6 AU/mL
Influenza Ab
100% = 26.0 U/mL
VZV Ab
100% = 46.0 ng/mL
Tet tox Ab
100% = 0.46 IU/mL
C
%of pre-cladribine B or T cells
Day
CD19+ B Cells
100% = 132767 cells/mL
CD3+ T Cells
100% = 451136 cells/mL
D
% of pre-cladribine CD4+ T cells
Day
Naive CD4+
100% = 163492 cells/mL
CD4+ TCM
100% = 29314 cells/mL
CD4+ TEM
100% = 60941 cells/mL
CD4+ TEMRA
100% = 156933 cells/mL
E
%of pre-cladribine CD8+ T cells
Day
Naive CD8+
100% = 13058 cells/mL
CD8+ TCM
100% = 598 cells/mL
CD8+ TEM
100% = 2545 cells/mL
CD8+ TEMRA
100% = 24256 cells/mL

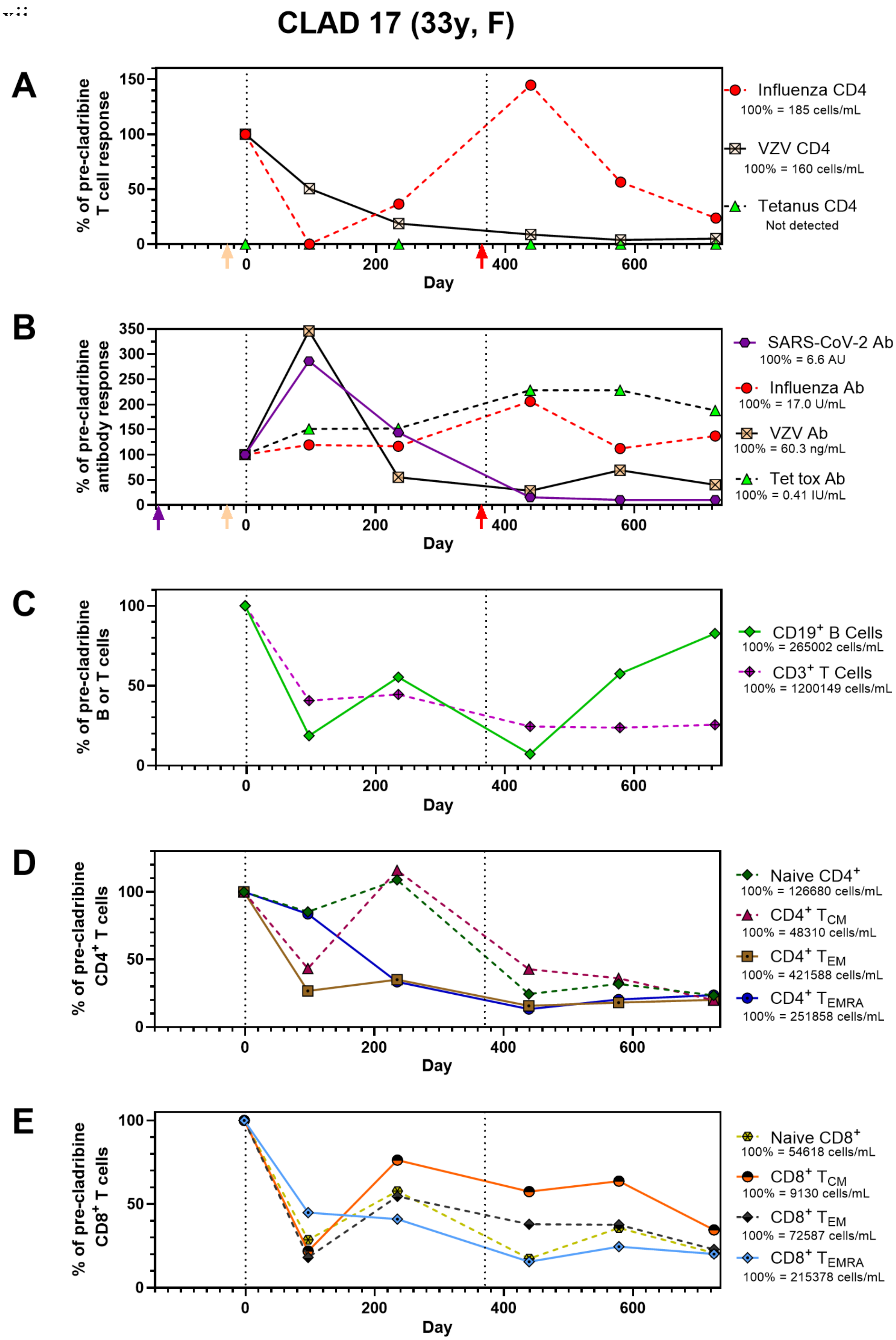
CLAD 17 (33y, F)
A
% of pre-cladribine T cell response
Day
Influenza CD4
100% = 185 cells/mL
VZV CD4
100% = 160 cells/mL
Tetanus CD4
Not detected
B
% of pre-cladribine antibody response
Day
SARS-CoV-2 Ab
100% = 6.6 AU
Influenza Ab
100% = 17.0 U/mL
VZV Ab
100% = 60.3 ng/mL
Tet tox Ab
100% = 0.41 IU/mL
C
% of pre-cladribine B or T cells
Day
CD19+ B Cells
100% = 265002 cells/mL
CD3+ T Cells
100% = 1200149 cells/mL
D
% of pre-cladribine CD4+ T cells
Day
Naive CD4+
100% = 126680 cells/mL
CD4+ TCM
100% = 48310 cells/mL
CD4+ TEM
100% = 421588 cells/mL
CD4+ TEMRA
100% = 251858 cells/mL
E
% of pre-cladribine CD8+ T cells
Day
Naive CD8+
100% = 54618 cells/mL
CD8+ TCM
100% = 9130 cells/mL
CD8+ TEM
100% = 72587 cells/mL
CD8+ TEMRA
100% = 215378 cells/mL

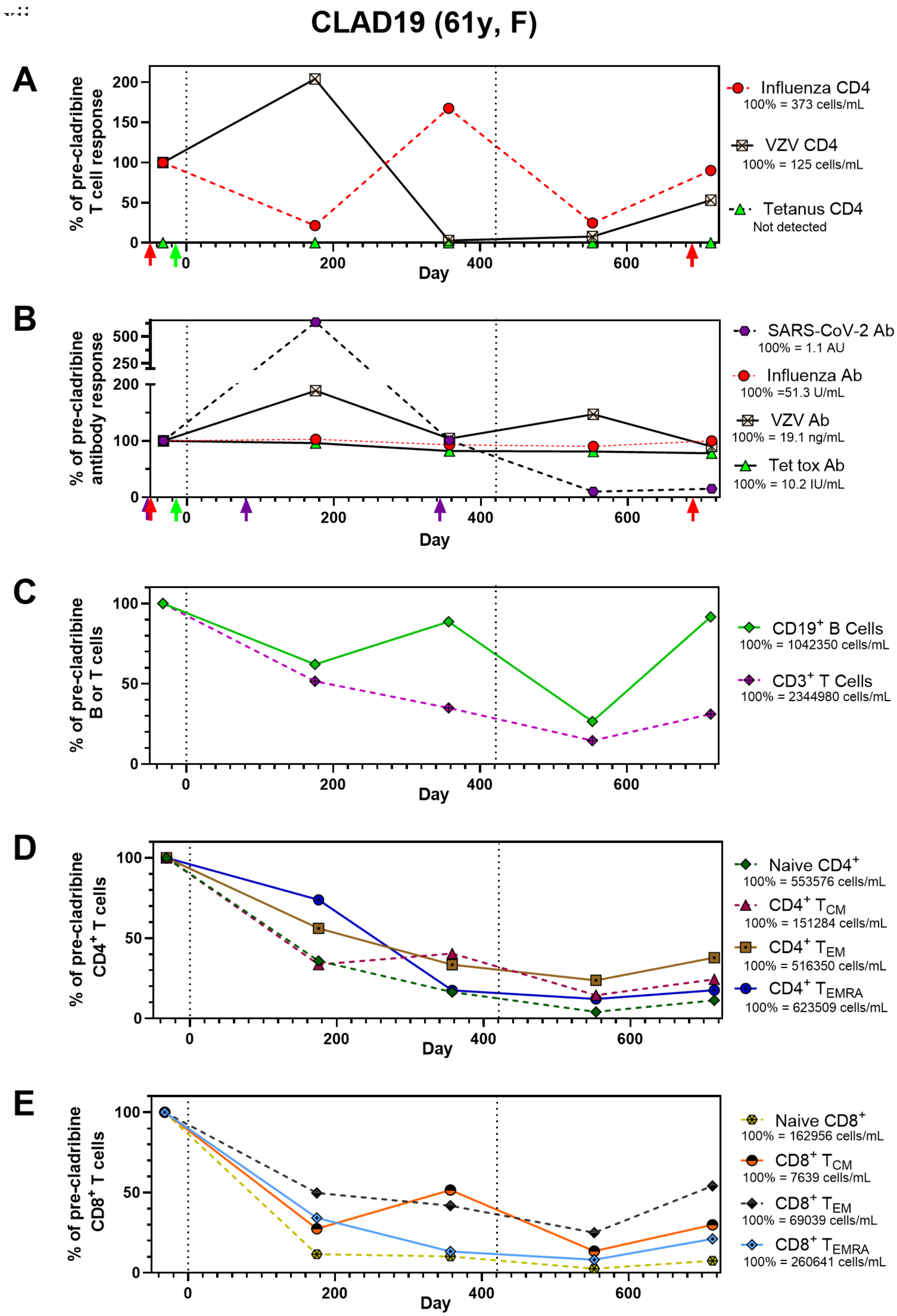
CLAD19 (61y, F)
A
% of pre-cladribine T cell response
Day
Influenza CD4
100% = 373 cells/mL
VZV CD4
100% = 125 cells/mL
Tetanus CD4
Not detected
B
% of pre-cladribine antibody response
Day
SARS-CoV-2 Ab
100% = 1.1 AU
Influenza Ab
100% =51.3 U/mL
VZV Ab
100% = 19.1 ng/mL
Tet tox Ab
100% = 10.2 IU/mL
C
% of pre-cladribine B or T cells
Day
CD19+ B Cells
100% = 1042350 cells/mL
CD3+ T Cells
100% = 2344980 cells/mL
D
% of pre-cladribine CD4+ T cells
Day
Naive CD4+
100% = 553576 cells/mL
CD4+ TCM
100% = 151284 cells/mL
CD4+ TEM
100% = 516350 cells/mL
CD4+ TEMRA
100% = 623509 cells/mL
E
% of pre-cladribine CD8+ T cells
Day
Naive CD8+
100% = 162956 cells/mL
CD8+ TCM
100% = 7639 cells/mL
CD8+ TEM
100% = 69039 cells/mL
CD8+ TEMRA
100% = 260641 cells/mL

CLAD20 (59y, M)

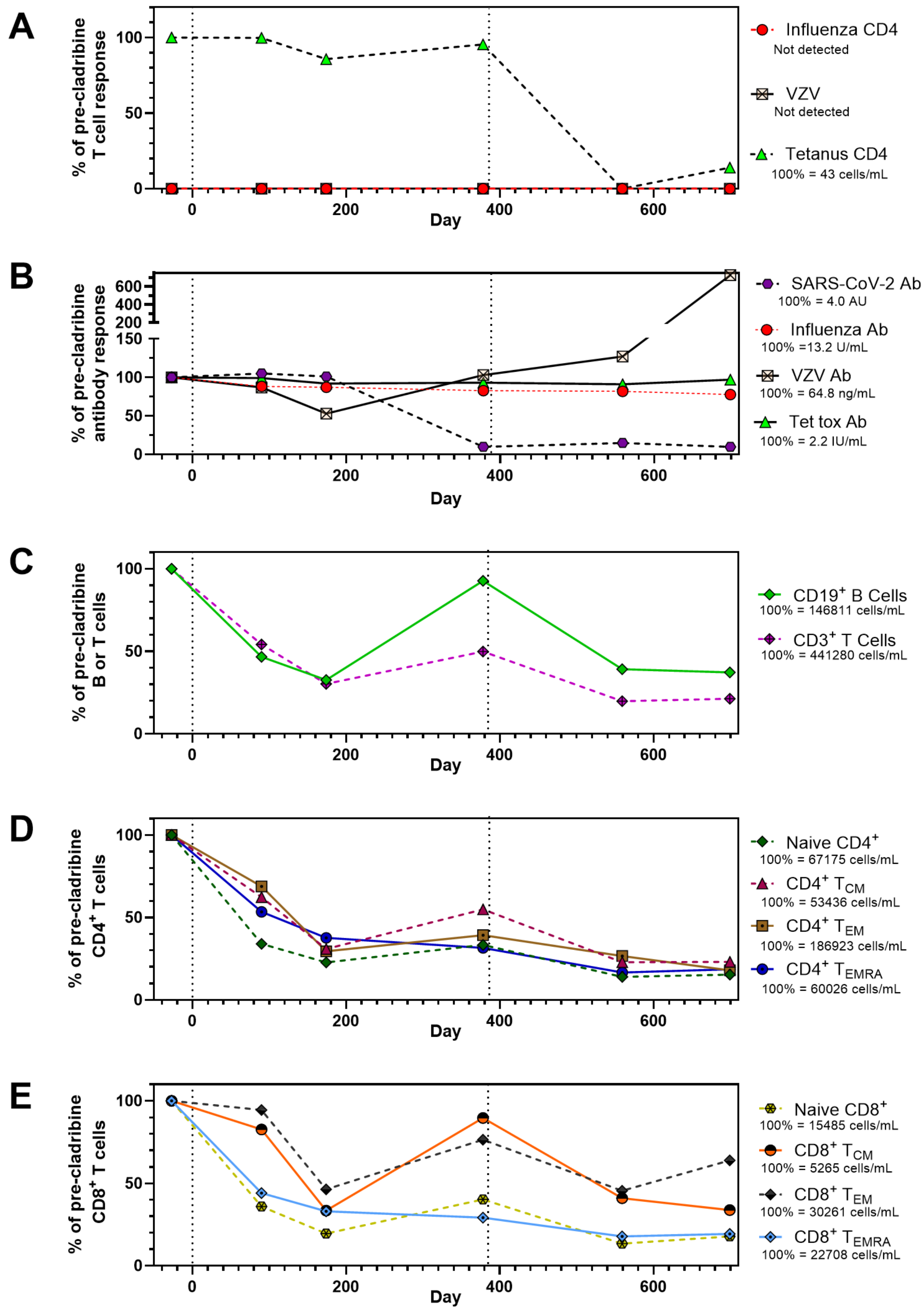
A
% of pre-cladribine T cell response
Day
Influenza CD4
Not detected
VZV
Not detected
Tetanus CD4
100% = 43 cells/mL
B
% of pre-cladribine antibody response
Day
SARS-CoV-2 Ab
100% = 4.0 AU
Influenza Ab
100% =13.2 U/mL
VZV Ab
100% = 64.8 ng/mL
Tet tox Ab
100% = 2.2 IU/mL
C
% of pre-cladribine B or T cells
Day
CD19+ B Cells
100% = 146811 cells/mL
CD3+ T Cells
100% = 441280 cells/mL
D
% of pre-cladribine CD4+ T cells
Day
Naive CD4+
100% = 67175 cells/mL
CD4+ TCM
100% = 53436 cells/mL
CD4+ TEM
100% = 186923 cells/mL
CD4+ TEMRA
100% = 60026 cells/mL
E
% of pre-cladribine CD8+ T cells
Day
Naive CD8+
100% = 15485 cells/mL
CD8+ TCM
100% = 5265 cells/mL
CD8+ TEM
100% = 30261 cells/mL
CD8+ TEMRA
100% = 22708 cells/mL

CLAD21 (61y, F)

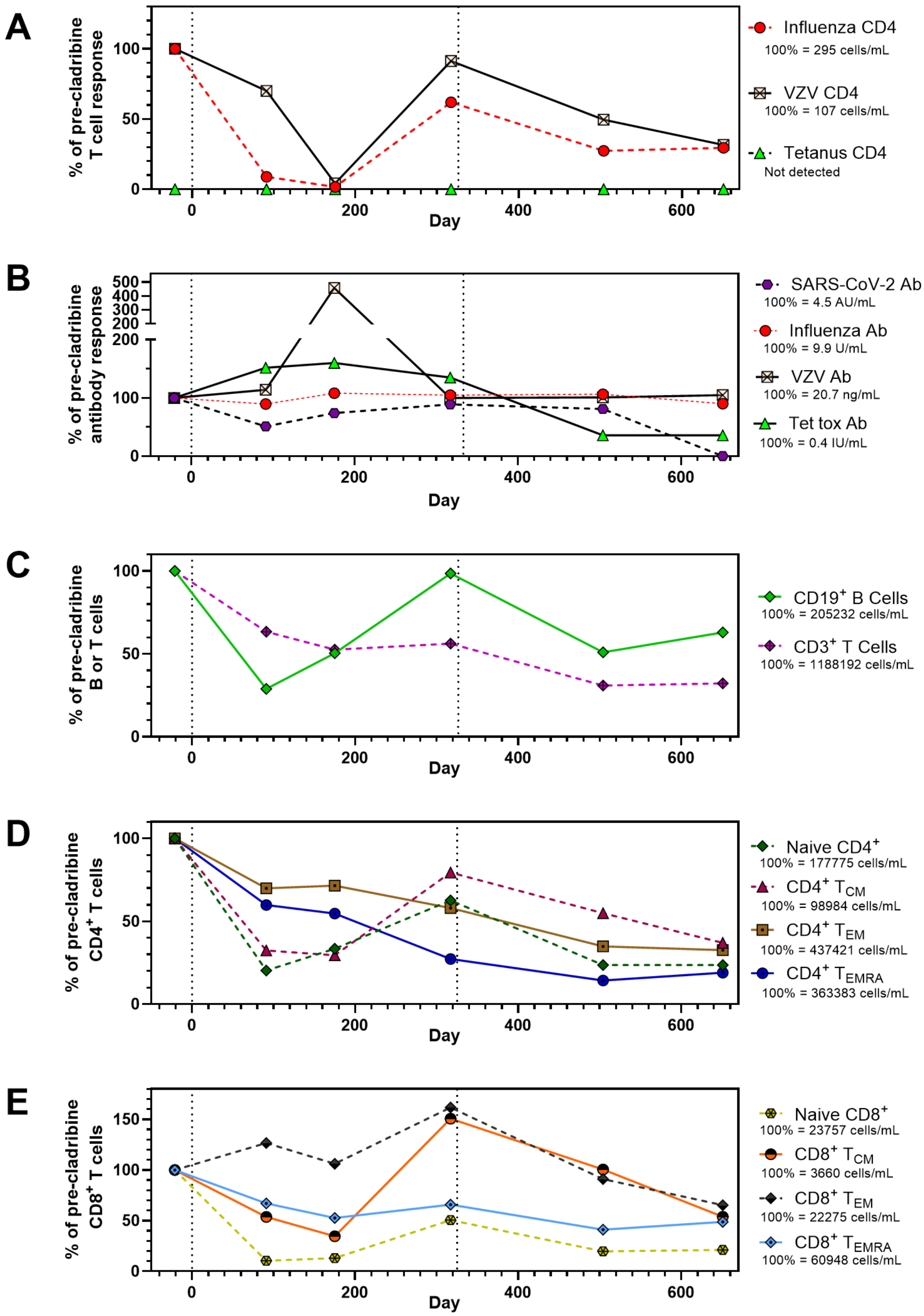
A
% of pre-cladribine T cell response
Day
Influenza CD4
100% = 295 cells/mL
VZV CD4
100% = 107 cells/mL
Tetanus CD4
Not detected
B
% of pre-cladribine antibody response
Day
SARS-CoV-2 Ab
100% = 4.5 AU/mL
Influenza Ab
100% = 9.9 U/mL
VZV Ab
100% = 20.7 ng/mL
Tet tox Ab
100% = 0.4 IU/mL
C
% of pre-cladribine B or T cells
Day
CD19+ B Cells
100% = 205232 cells/mL
CD3+ T Cells
100% = 1188192 cells/mL
D
% of pre-cladribine CD4+ T cells
Day
Naive CD4+
100% = 177775 cells/mL
CD4+ TCM
100% = 98984 cells/mL
CD4+ TEM
100% = 437421 cells/mL
CD4+ TEMRA
100% = 363383 cells/mL
E
% of pre-cladribine CD8+ T cells
Day
Naive CD8+
100% = 23757 cells/mL
CD8+ TCM
100% = 3660 cells/mL
CD8+ TEM
100% = 22275 cells/mL
CD8+ TEMRA
100% = 60948 cells/mL

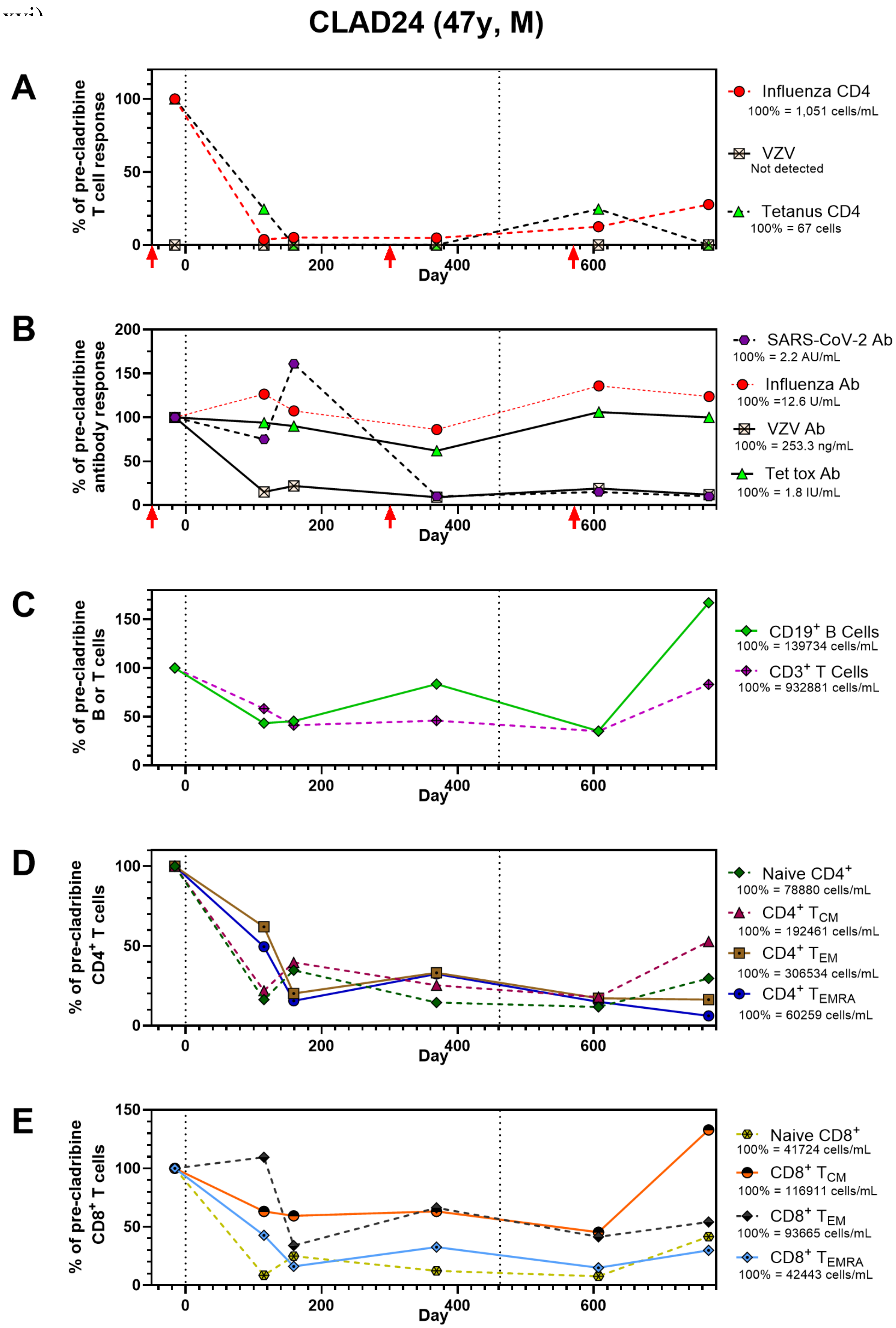
CLAD24 (47y, M)
A
% of pre-cladribine T cell response
Day
Influenza CD4
100% = 1,051 cells/mL
VZV
Not detected
Tetanus CD4
100% = 67 cells
B
% of pre-cladribine antibody response
Day
SARS-CoV-2 Ab
100% = 2.2 AU/mL
Influenza Ab
100% =12.6 U/mL
VZV Ab
100% = 253.3 ng/mL
Tet tox Ab
100% = 1.8 IU/mL
C
% of pre-cladribine B or T cells
Day
CD19+ B Cells
100% = 139734 cells/mL
CD3+ T Cells
100% = 932881 cells/mL
D
% of pre-cladribine CD4+ T cells
Day
Naive CD4+
100% = 78880 cells/mL
CD4+ TCM
100% = 192461 cells/mL
CD4+ TEM
100% = 306534 cells/mL
CD4+ TEMRA
100% = 60259 cells/mL
E
% of pre-cladribine CD8+ T cells
Day
Naive CD8+
100% = 41724 cells/mL
CD8+ TCM
100% = 116911 cells/mL
CD8+ TEM
100% = 93665 cells/mL
CD8+ TEMRA
100% = 42443 cells/mL

CLAD26 (44y, F)

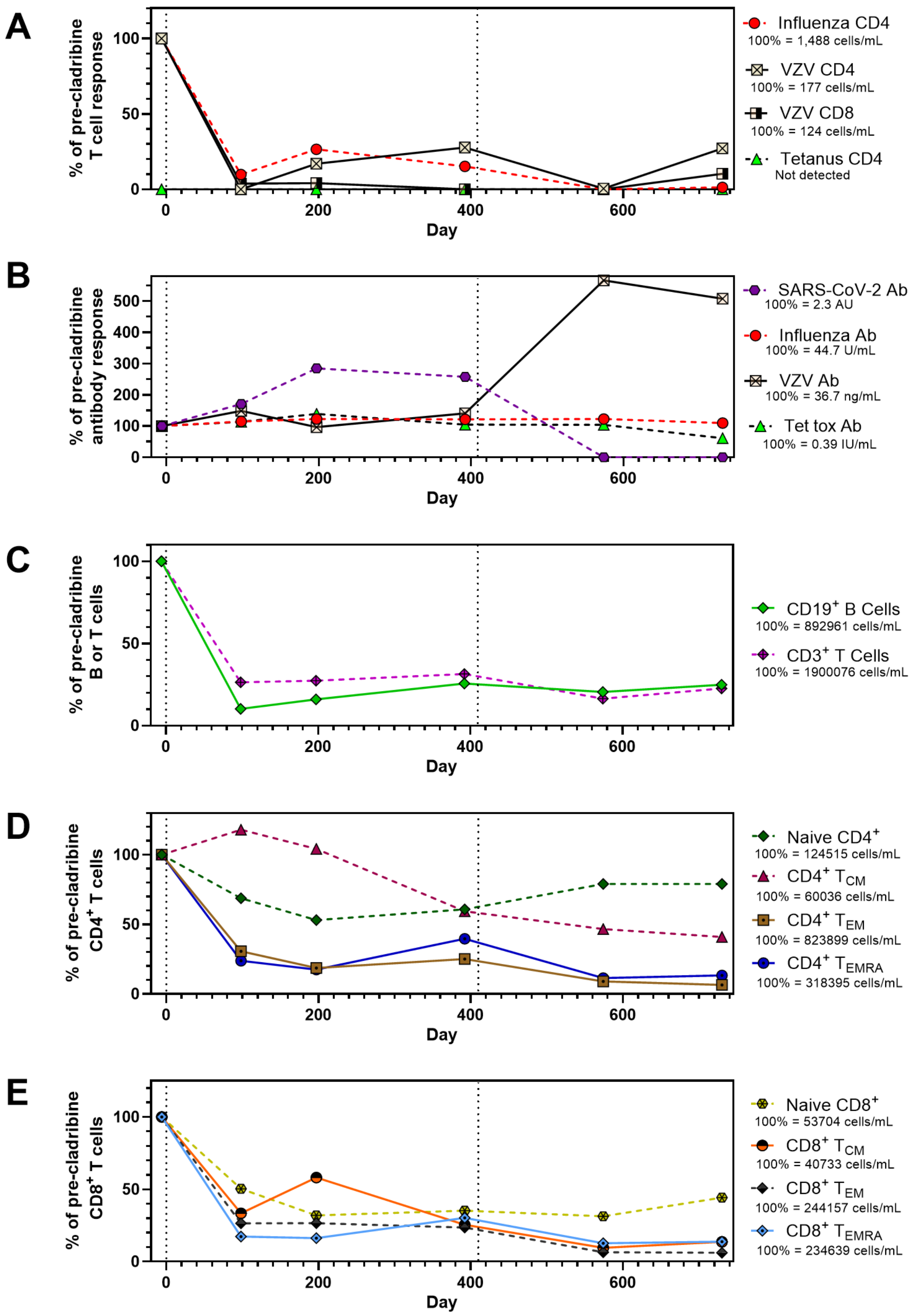
A
% of pre-cladribine T cell response
Day
Influenza CD4
100% = 1,488 cells/mL
VZV CD4
100% = 177 cells/mL
VZV CD8
100% = 124 cells/mL
Tetanus CD4
Not detected
B
% of pre-cladribine antibody response
Day
SARS-CoV-2 Ab
100% = 2.3 AU
Influenza Ab
100% = 44.7 U/mL
VZV Ab
100% = 36.7 ng/mL
Tet tox Ab
100% = 0.39 IU/mL
C
% of pre-cladribine B or T cells
Day
CD19^{+} B Cells
100% = 892961 cells/mL
CD3^{+} T Cells
100% = 1900076 cells/mL
D
% of pre-cladribine CD4^{+} T cells
Day
Naive CD4^{+}
100% = 124515 cells/mL
CD4^{+} T_{CM}
100% = 60036 cells/mL
CD4^{+} T_{EM}
100% = 823899 cells/mL
CD4^{+} T_{EMRA}
100% = 318395 cells/mL
E
% of pre-cladribine CD8^{+} T cells
Day
Naive CD8^{+}
100% = 53704 cells/mL
CD8^{+} T_{CM}
100% = 40733 cells/mL
CD8^{+} T_{EM}
100% = 244157 cells/mL
CD8^{+} T_{EMRA}
100% = 234639 cells/mL

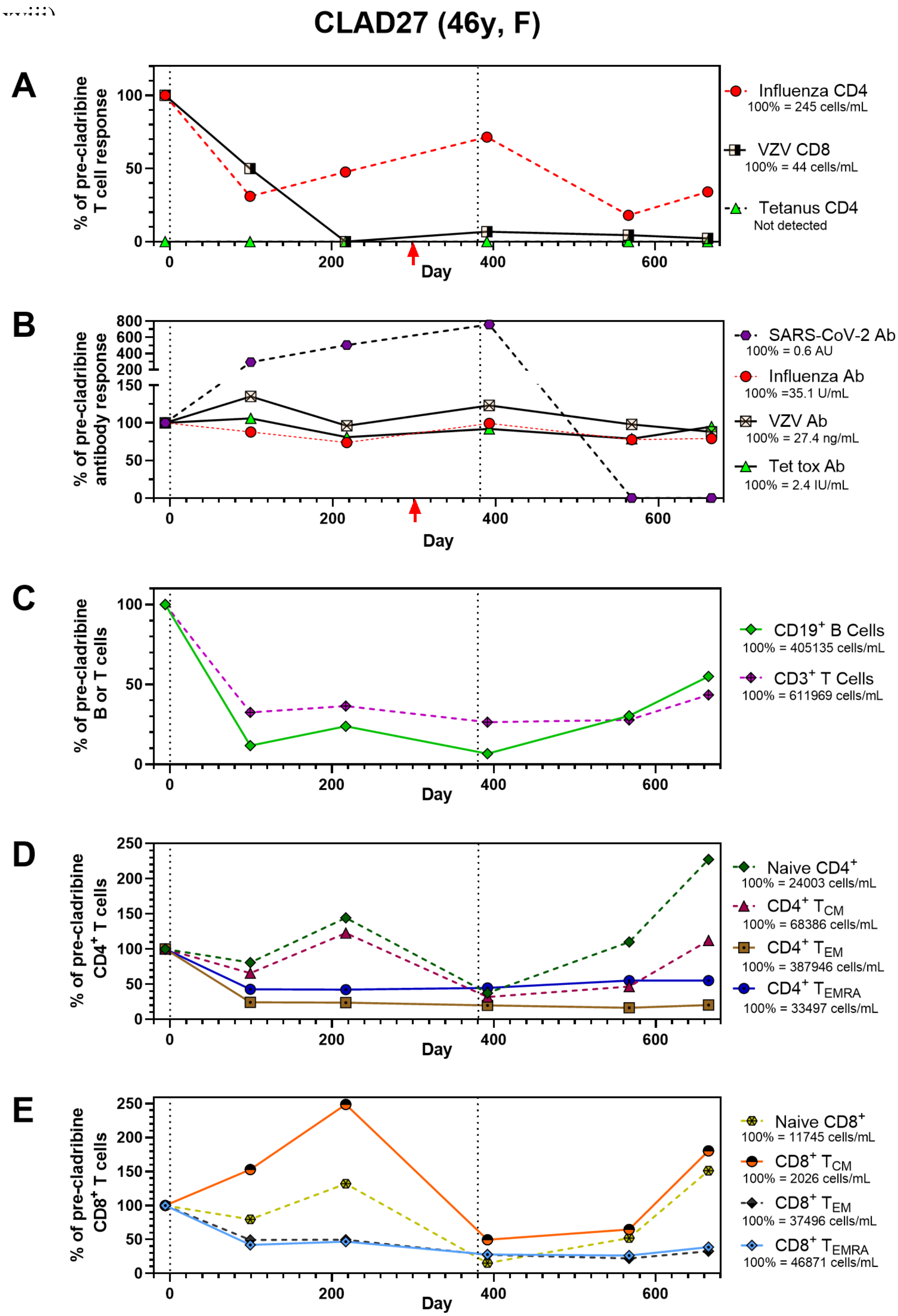
CLAD27 (46y, F)
A
% of pre-cladribine T cell response
Day
Influenza CD4
100% = 245 cells/mL
VZV CD8
100% = 44 cells/mL
Tetanus CD4
Not detected
B
% of pre-cladribine antibody response
Day
SARS-CoV-2 Ab
100% = 0.6 AU
Influenza Ab
100% =35.1 U/mL
VZV Ab
100% = 27.4 ng/mL
Tet tox Ab
100% = 2.4 IU/mL
C
% of pre-cladribine B or T cells
Day
CD19+ B Cells
100% = 405135 cells/mL
CD3+ T Cells
100% = 611969 cells/mL
D
% of pre-cladribine CD4+ T cells
Day
Naive CD4+
100% = 24003 cells/mL
CD4+ TCM
100% = 68386 cells/mL
CD4+ TEM
100% = 387946 cells/mL
CD4+ TEMRA
100% = 33497 cells/mL
E
% of pre-cladribine CD8+ T cells
Day
Naive CD8+
100% = 11745 cells/mL
CD8+ TCM
100% = 2026 cells/mL
CD8+ TEM
100% = 37496 cells/mL
CD8+ TEMRA
100% = 46871 cells/mL

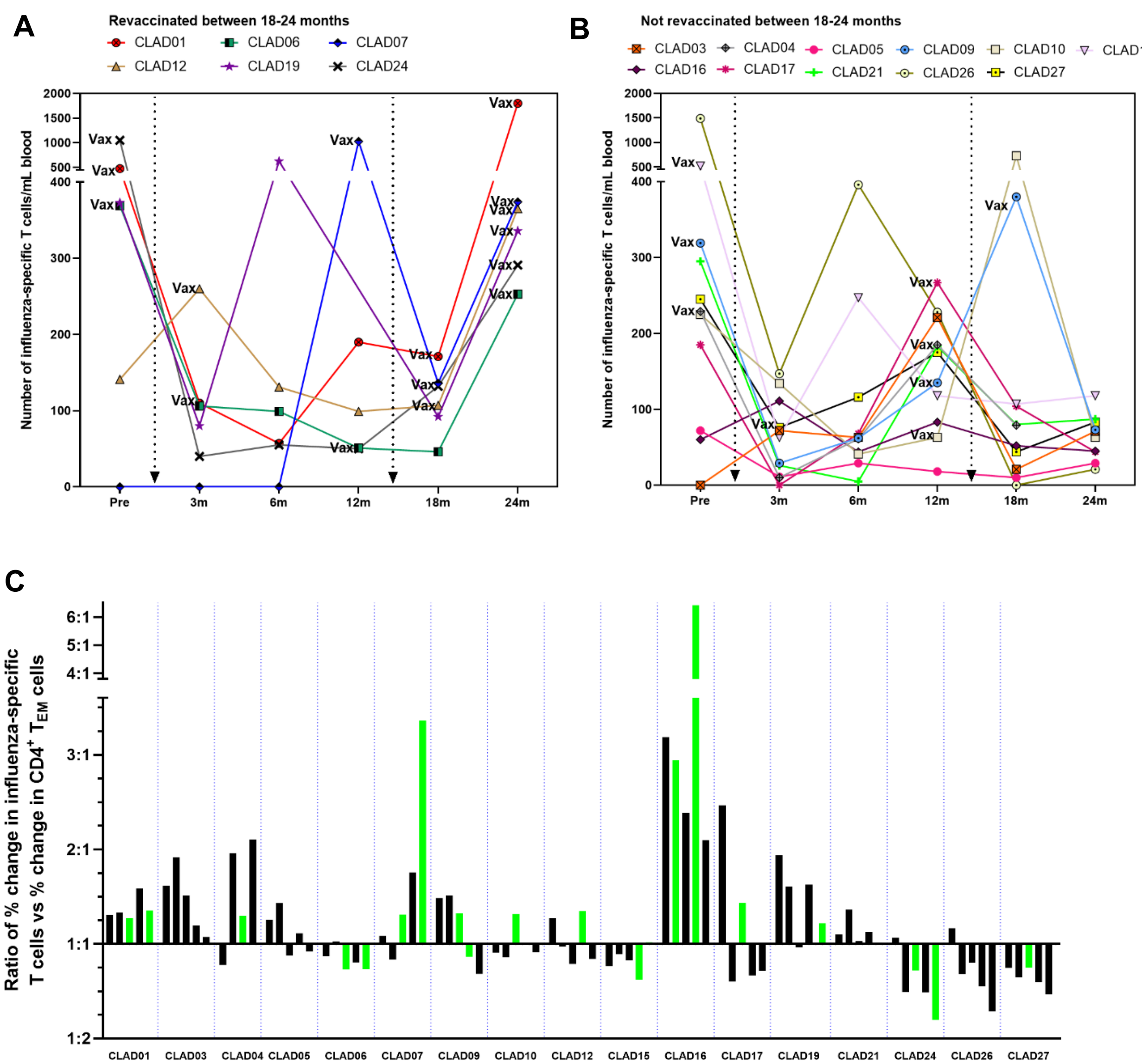

Supplementary Figure S3. **A & B)** Changes in numbers of influenza-specific T cells over time: influence of timing of re-vaccinations on outcome after CladT treatment. "Vax" to the left of a symbol indicates that the patient was re-vaccinated less than 150 days before that specific blood sample was taken. The vertical dotted lines represent the two treatments with CladT. **A)** Pattern of responses in patients who were re-vaccinated between months 18 and 24 after initial dose of CladT and **B)** Pattern of responses in people who were not re-vaccinated between months 18-24. **C)** Changes in numbers of influenza-specific $CD4^+$ T cells are independent of changes in numbers of $CD4^+$ T_{EM} cells in many patients. Each bar shows the ratio of the % change from baseline of the influenza-specific cells to the % change from baseline of the total $CD4^+$ T_{EMRA} cells at each timepoint. If the influenza-specific cells only changed due to changes in total numbers of $CD4^+$ T_{EMRA} cells, then the bar would be at the line at 1:1. Green bars represent timepoints where there had been a vaccination just prior to that timepoint. Statistical analysis: Friedman test with correction for multiple comparisons by controlling the false discovery rate (overall $P<0.008$, indicating that the typical patient does not stay at a 1:1 ratio).

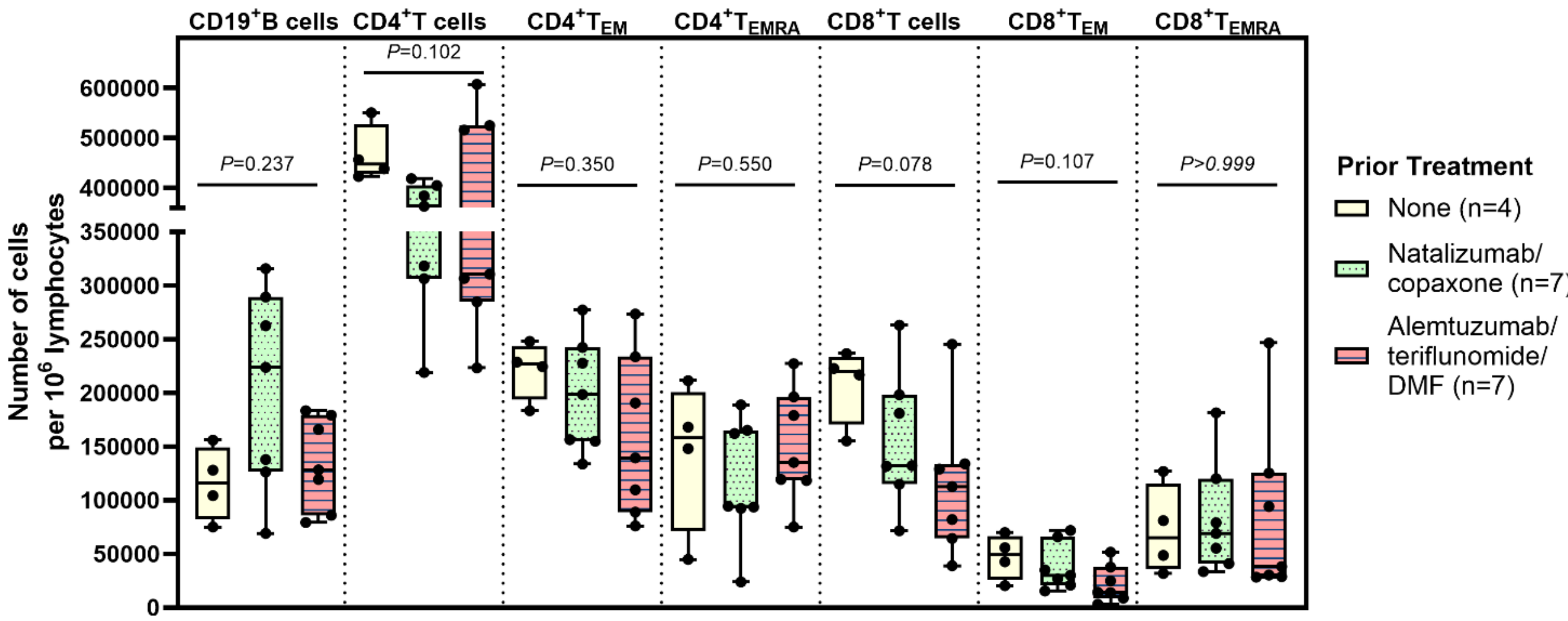

Supplementary Figure S4. Effects of prior disease modifying therapies (DMTs) on lymphocyte subsets. The number of each lymphocyte subset per 10^6 lymphocytes, grouped by whether patients had no prior DMT exposure (n=4), had been treated with natalizumb or copaxone (which do not have long-term impacts on lymphocytes; n=7) or with alemtuzumab, teriflunomide or dimethyl fumarate (DMF) (all of which can have continuing effects on lymphocyte biology for several years; n=7). Statistical analysis: Friedman test with Dunn's correction for multiple comparisons. The overall Friedman test P value is shown in the figure. Only the $CD8^+$ T cell subset approached significance.

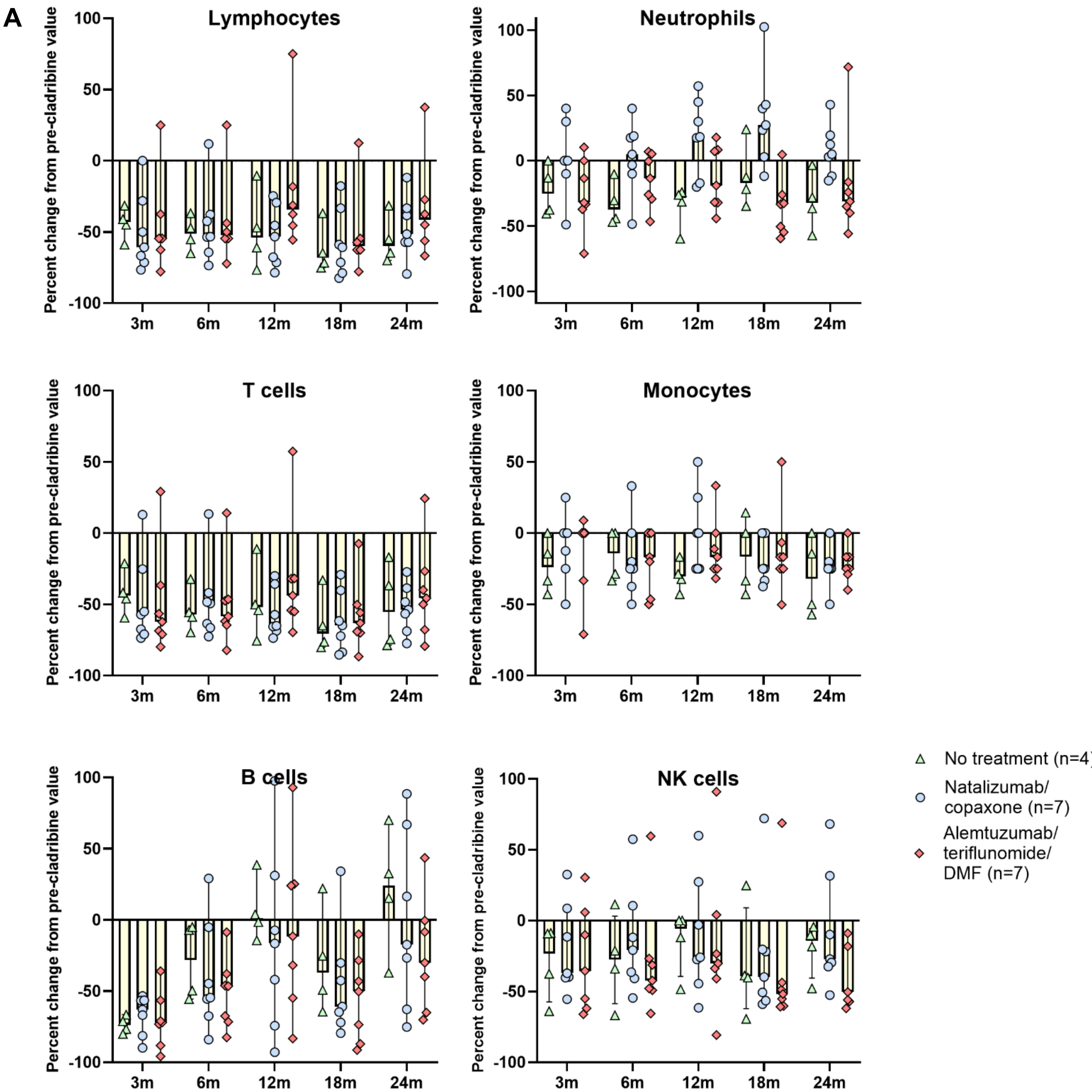

Supplementary Figure S5. (A) Breakdown of data from Figure 1, based on prior DMT treatment. There were no significant differences between groups when analyzed using the Friedman test, although the low number of individuals in each group and the variation between individual patients makes the power of this statistical test low. (B) – graph shown on next page. Breakdown of data from Figure 2, based on prior DMT treatment. The limitations for the analysis are the same as in Figure A.

B

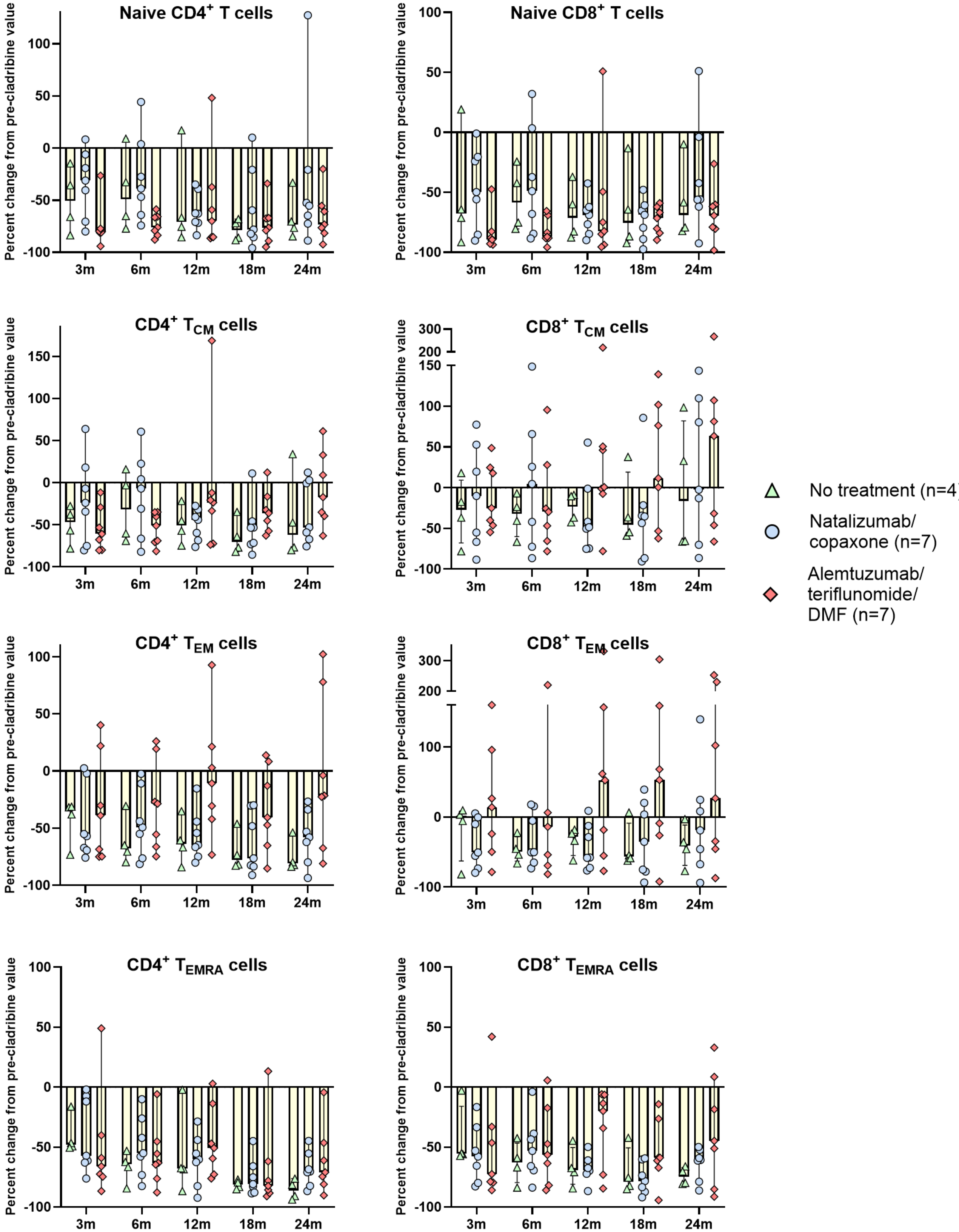
Naive CD4+ T cells
Naive CD8+ T cells
CD4+ TCM cells
CD8+ TCM cells
CD4+ TEM cells
CD8+ TEM cells
CD4+ TEMRA cells
CD8+ TEMRA cells
Percent change from pre-cladribine value
3m
6m
12m
18m
24m
No treatment (n=4)
Natalizumab/ copaxone (n=7)
Alemtuzumab/ teriflunomide/ DMF (n=7)
